# A genome-wide association study identifies that *SAMD5* interacts with regular Sun exposure to influence nasolabial folds development

**DOI:** 10.1186/s40101-026-00423-z

**Published:** 2026-02-28

**Authors:** Jun Yan Ng, Yang Yie Sio, Fook Tim Chew

**Affiliations:** 1 Department of Biological Sciences, Faculty of Science, National University of Singapore, Singapore, 117543 Singapore; 2 Department of Biological Sciences, Faculty of Science, National University of Singapore, Allergy and Molecular Immunology Laboratory, Lee Hiok Kwee Functional Genomics Laboratories, Block S2, Level 5, 14 Science Drive 4, Lower Kent Ridge Road, Singapore, 117543 Singapore

**Keywords:** Skin, Ageing, Aging, Epidemiology, Genetics, Nasolabial folds, Wrinkles, *SAMD5*, Sun exposure, Skin type

## Abstract

**Background:**

Skin ageing is influenced by genetics, chronological age, and Sun exposure. Nasolabial folds are wrinkles prevalent among young ethnic Chinese participants in the Singapore/Malaysia Cross-sectional Genetics Epidemiology Study (SMCGES).

**Methods:**

We analysed data from 4421 SMCGES ethnic Chinese young adults. Collected data included demographics, Sun exposure, Fitzpatrick Skin Type, and nasolabial fold presence, assessed using validated questionnaires and photo-numeric scales. Genetic data were obtained through SNP genotyping, imputation, and whole transcriptome sequencing. Buccal cell samples from 2776 participants across three sites (National University of Singapore [NUS], University of Tunku Abdul Rahman [UTAR], and Sunway University [SU]) were used for SNP genotyping, and peripheral blood mononuclear cell samples from 658 participants at NUS and UTAR for sequencing. Analyses were performed using HaploView, RStudio, and PLINK, integrating data from the Genotype-Tissue Expression (GTEx) portal, eQTLGen consortium, and NCBI Gene Expression Omnibus.

**Results:**

Our GWAS identified *SAMD5* as associated with nasolabial folds among individuals with regular Sun exposure. *SAMD5* SNPs might modulate binding of microRNAs hsa-miR-216a and hsa-miR-485-5p, suppressing *SAMD5* expression and promoting nasolabial folds. rs844607 increased odds of nasolabial folds (AOR = 2.67 [1.89–3.77], *p* = 2.27 × 10^−8^) and forms a risk haplotype with 3′-end SNPs predicted as miRNA binding sites. The likely causal SNP, rs702344, strengthens miRNA binding (hsa-miR-216a: score 151, ΔG = − 19.64 kcal/mol; hsa-miR-485-5p: score 157, ΔG = − 22.36 kcal/mol), suppressing *SAMD5* expression. eQTL data from GTEx (NES = − 0.72, *p* = 2.79 × 10^−52^) and eQTLGen (*Z* = 6.16, *p* = 6.59 × 10^−6^) supported this model. Lower *SAMD5* expression was observed among chronologically aged individuals (GEO GSE200002, logFC = − 0.639, adj. *p *= 7.57 × 10^−3^) and in untreated photo-aged dermal fibroblasts relative to retinoid-treated ones (GEO GSE294121, adj. *p* = 3.00 × 10^−2^). *SAMD5* promotes melanogenesis and UV protection; t-allele carriers showed 1.6-fold higher odds of melanin-poor burning skin types (95% CI 1.11–2.28; *p* = 0.012). Regular Sun exposure increased odds of nasolabial folds 1.26-fold (95% CI 1.08–1.46; *p* = 0.0028), whereas melanin-rich tanning skin types reduced the odds (AOR = 0.68; 95% CI 0.47–0.98; *p* = 0.048).

**Conclusion:**

These findings support a model where rs702344 enhances miRNA binding, downregulates *SAMD5*, reduces melanogenesis, and promotes nasolabial folds in Sun-exposed young adults.

**Supplementary Information:**

The online version contains supplementary material available at 10.1186/s40101-026-00423-z.

## Introduction

Skin ageing is defined as any change to the skin that occurs due to ageing [1] and manifests on the skin in at least 56 described forms [2]. While skin ageing is inevitable, its pace varies among individuals due to differences in intrinsic factors (e.g., genetic factors, chronological influences) [2] and extrinsic factors (environmental sources) [3]. We reported in previous meta-analyses and systematic reviews that age, sex, race, nutrition, smoking, air pollution, and Sun exposure (i.e., exposure to ultraviolet (UV) light from the Sun), and genetics [2, 4] are key risk factors driving the development of skin ageing phenotypes [1].

Nasolabial folds are a distinctive and clinically recognisable feature of skin ageing, important for three reasons. First, they are an early-onset ageing phenotype, appearing in individuals younger than 30 years of age [5]. Second, our previous analysis of the Singapore/Malaysia Cross-sectional Genetics Epidemiology Study (SMCGES) participants mostly in their mid-20s showed that nasolabial folds are present in 54% of ethnic Chinese participants [6]. This high prevalence provides strong statistical justification for detailed analyses. Third, our earlier Principal Component Analysis (PCA) publication demonstrated that nasolabial folds are highly concordant with the first principal component (PC1) of skin ageing phenotypes in the SMCGES [6]. As PC1 captures the largest source of variability, nasolabial folds is a representative phenotype describing skin ageing within the SMCGES.

Through a genome-wide association study (GWAS), we found that *SAMD5* is associated with the development of nasolabial folds. Here, we aim to understand how the interaction between *SAMD5* and Sun exposure leads to nasolabial folds. We offer a plausible mechanism of action and hypothesise that SNPs in the *SAMD5* locus affect the binding of microRNA (miRNA), thereby modulating *SAMD5* expression and recapitulating this facial wrinkling phenotype.

## Methodology

### Study design and participant recruitment

We introduced the SMCGES in our earlier works [7–14]. Briefly, the SMCGES is an ongoing genetics and epidemiology collection conducted in Singapore and Malaysia universities. Volunteers were recruited from 2011 to 2025 from Singapore (National University of Singapore (NUS)) and Malaysia (Sunway University (SU) and University of Tunku Abdul Rahman (UTAR)) universities via walk-in and their skin were assessed for signs of skin ageing (Table 1). No volunteers were excluded from the study, however, analysis for the current assessment is limited to SMCGES ethnic Chinese participants.

**Table 1:** Demographics of Singapore and Malaysia participants recruited from the Singapore/Malaysia Cross-sectional Genetics Epidemiology Study (SMCGES) for the current assessment

Demographics	Ethnic Chinese young adults (*n* = 4421)
Age, mean (SD), years	25.8 ± 7.2^a^
Height, mean (SD), cm	165.3 ± 8.3^a^
Weight, mean (SD), kg	60.0 ± 12.8^a^
BMI, mean (SD), kg/m^2^	21.9 ± 3.8^a^
Sex^b^	
Male	1471 (33%)
Female	2950 (67%)
Housing	
HDB flat/flat	2133 (48%)^c^
Condominium/private apartment	1155 (26%)^c^
Landed property	1125 (25%)^c^
Missing/invalid^d^	8 (< 1%)^c^
Nasolabial folds^e^	
Controls	3856 (87%)
Cases	565 (13%)
Regular Sun exposure^f^	
Absent	3065 (69%)
Present	1356 (31%)

*Abbreviations: SMCGES* Singapore/Malaysia Cross-sectional Genetics Epidemiology Study, *SD* standard deviation, *cm* centimetres, *kg* kilograms, *HDB flat* Singapore’s Housing Development Board flat, *BMI* body mass index, *kg/m*^*2*^ kilograms per square metre

^a^The values after ± are standard deviation values

^b^Sex was self-reported

^c^Percentages may not total 100 due to rounding

^d^Missing/Invalid referred to responses that were either left blank or otherwise invalid

^e^Of the genotyped participants, 2432 are controls and 344 are cases. Genotyping for the remaining participants, mostly participants recruited in 2024 and 2025, is ongoing

^f^Of the genotyped participants, 1889 are controls and 887 are cases. Genotyping for the remaining participants, mostly participants recruited in 2024 and 2025, is ongoing. Sun exposure was quantified using a validated open-ended survey question on outdoor recreational activity frequency and duration by Morales-Sánchez et al. (2014)

Nasolabial folds were self-reported on a five-grade validated photo-numeric scale developed by Narins et al. (2012) [15]. Nasolabial folds matching the upper three grades of the photo-numeric scale are deemed as present. To account for phenotype ascertainment bias, we comparing different skin ageing evaluation methods and compared a randomised subset of self-reported phenotypes against independent assessor gradings [16–19]. Participants self-reported sex, race, housing, and Sun exposure through an investigator-administered questionnaire. Sun exposure was quantified using a validated open-ended survey question on the frequency and duration in which the participant practices any outdoor recreation activity [20]. Skin type (burn or tan) was quantified based on the Fitzpatrick Skin Type using a validated Sun exposure questionnaire [21].

### Collection of genetic data

Genetic data was collected in two ways. First, peripheral blood mononuclear cell (PBMC) samples of 658 participants were collected from NUS and UTAR for whole transcriptome sequencing and SNP genotyping assay. Second, buccal cell samples of *n* = 2776 participants of Chinese ethnicity were collected from NUS, UTAR, and SU for SNP genotyping assay.

### Whole-transcriptome sequencing

To perform the whole-transcriptome sequencing assay, 10 mL of whole blood was collected and PBMCs were extracted using the Ficoll-Hypaque density-gradient centrifugation approach. Total RNA was extracted from the isolated PBMCs using the E.Z.N.A.^®^ Total RNA Kit (Omega Bio-tek Inc., USA), following guidelines provided by the manufacturer. Enrichment of messenger RNA (mRNA) and library construction was performed using oligo(dT) beads and the NEBNext Ultra RNA Library Prep Kit according to the manufacturer’s protocols. All next-generation sequencing (NGS) sample preparation procedures using commercially available kits were performed following the manufacturer’s instructions. Next-generation sequencing was conducted on the Illumina NovaSeq 6000 platform. The generated paired end reads were mapped to the NCBI GRCh38 human reference genome assembly using TopHat version 2.1.1 to compute raw read counts.

These read counts were normalised to fragments per kilobase of transcript per million mapped reads (FPKM) with Cufflinks version 2.2.1. Transcripts per million (TPM) values were then obtained by dividing the FPKM of each gene by the sum of the FPKM across all genes in the sample. Finally, TPM values were normalised across samples through inverse normal transformation (INT), as outlined in the GTEx portal protocols. To minimise batch variation, all samples were sequenced simultaneously. Expression quantitative trait loci (eQTL) and differentially expressed genes (DEG) analysis for the SMCGES were conducted using this dataset. Genomic DNA (gDNA) was also extracted from PBMC using Axygen^®^ AxyPrep™ Multisource Genomic Miniprep DNA kit (Axygen, USA) for subsequent genotyping assay.

### Genotyping

SNP genotyping assay was performed on gDNA samples extracted from buccal cell samples and PBMC samples. Buccal cell samples were collected from participants by mouthwash using 10 mL of 0.9% saline. The gDNA samples were isolated from the buccal cell samples using the Axygen^®^ AxyPrep™ Multisource Genomic Miniprep DNA kit (Axygen, USA). DNA concentration was measured in triplicates using the NanoDrop 2000 spectrophotometer (Thermo Scientific, Singapore). Genotyping was performed using six GWAS arrays (Infinium OmniZhongHua-8 v1.3 BeadChip platform, Illumina HumanHap 550 k BeadChip version 3, InfiniumOmni2-5Exome, Infinium Global Screening Array [Illumina, USA], Infinium OmniZhonghua-8 v1.4 BeadChip platform, and Infinium Asian Screening Array v1.0). Haplotype phasing and data imputations were conducted using the IMPUTE2 v2.0 software with information from the 1000 Genomes Project (1kGP) phase I Beijing Han Chinese (CHB) database. The imputed data from all six arrays were consolidated for downstream analysis.

### Statistical analysis

miRNA binding information was obtained using the miRanda bioinformatic algorithm available on SNPinfo (https://snpinfo.niehs.nih.gov/snpinfo/snpfunc.html). The linkage disequilibrium (LD) pattern and *R*-square values between SNPs were calculated using two independent datasets: the 1kGP CHB dataset and the SMCGES dataset and visualised using Haploview^®^ ver4.2 (http://www.broadinstitute.org/haploview).

The chi-square exact test for Hardy–Weinberg equilibrium (HWE), logistic regression test (age- and sex-adjusted) for genotype–phenotype associations, and odds ratios (OR) were computed using the PLINK ver. 1.90. Genotype–phenotype associations with nasolabial folds was tested by a Genome-wide Association Study (GWAS) using a logistic regression model assuming an additive allele effect with adjustment to age, sex, and the top three genetic principal components to correct for possible population stratification. The GWAS significant threshold was defined as *p* = 5 × 10^–8^. Manhattan plots and quantile–quantile plots (QQ plot) were generated using the qqman package on R with the RStudio interface (R Foundation for Statistical Computing, 2010) in RStudio version 4.3.1 (R Foundation for Statistical Computing, Vienna, Austria).

Univariate and multivariate exposure-phenotype logistic regression tests were computed on R with the same RStudio interface using the same R program version and visualised in Microsoft Excel. Chi-square tests and chi-square tests for trend for exposure-phenotype associations were computed in Epi Info (https://www.cdc.gov/epiinfo/index.html) and visualised in Microsoft Excel.

## Results

### *SAMD5* is associated with nasolabial folds

We identified a significant association between the *SAMD5* locus and nasolabial folds, but only among participants with regular Sun exposure. This association was not observed in either the non-Sun-exposed or the unstratified groups (Additional files 1–6).

**Fig. 1:**
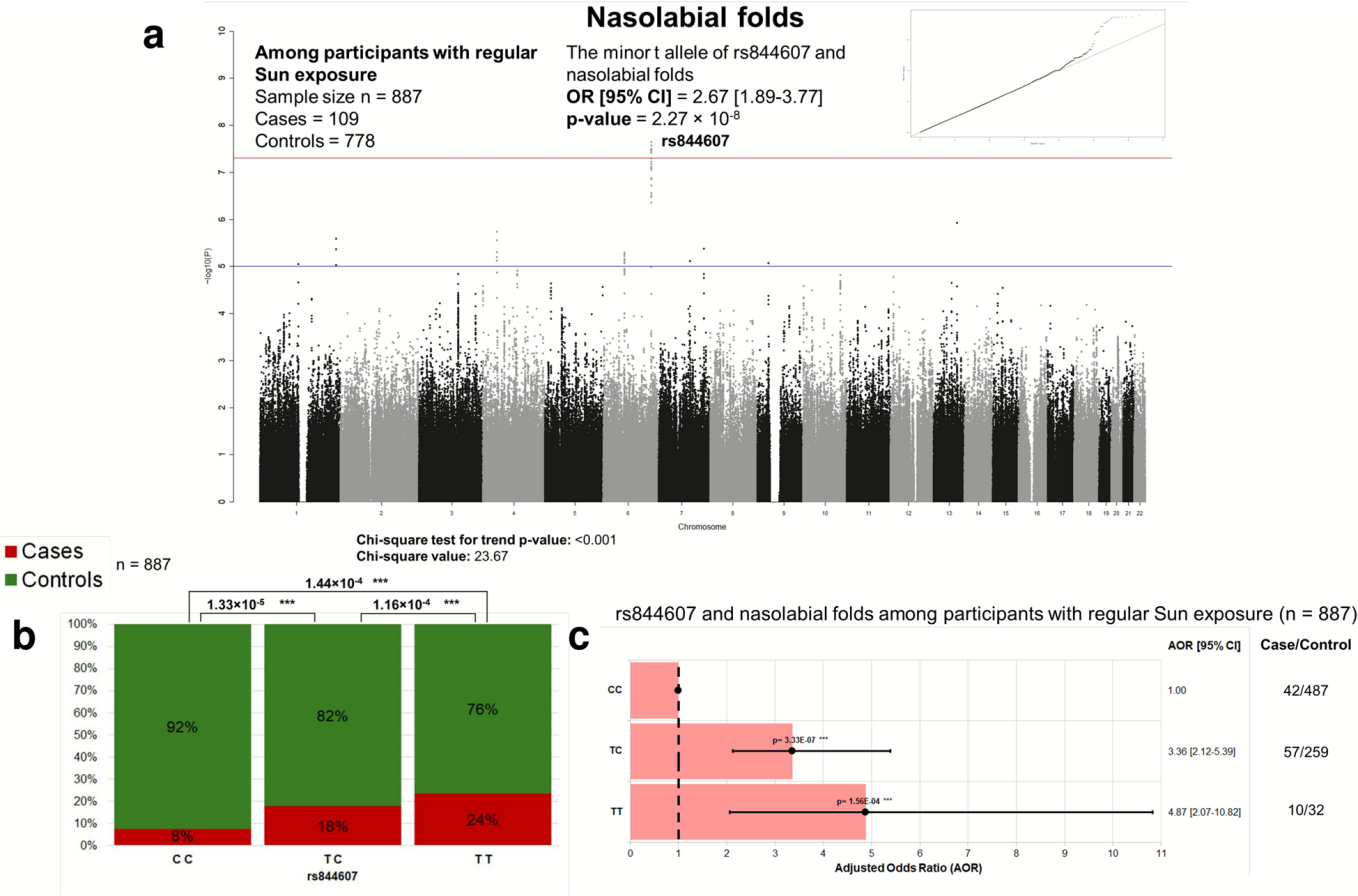
**a** Genome-wide association study (GWAS) of nasolabial folds in participants with regular Sun exposure from the Singapore/Malaysia Cross-sectional Genetics Epidemiology Study (SMCGES). This Manhattan plot, generated using the qqman package on R with the RStudio interface in RStudio version 4.3.1, shows the genome-wide distribution of single nucleotide polymorphism (SNP) associations with nasolabial folds among participants reporting regular Sun exposure (*n* = 887; cases = 109, controls = 778). Each point represents an SNP, with chromosomal position on the *x*-axis and the corresponding –log_10_(*p*-value) on the *y*-axis. The blue horizontal line denotes the suggestive significance threshold (*p* = 1 × 10^−5^), while the red line marks the genome-wide significance threshold (*p *= 5 × 10^−8^). A clear drag of association signals is observed on chromosome 6 near the *SAMD5* locus, with the lead SNP rs844607 exhibiting the strongest association; the minor t allele is associated with nasolabial folds, with an adjusted odds ratio (AOR) [95% confidence interval (CI)] = 2.67 [1.89–3.77], *p* = 2.27 × 10^−8^. The GWAS included 3,565,291 SNP variants genotyped from buccal cell DNA of 2776 Chinese participants, with a total genotyping rate of 0.981549. The analysis was adjusted for age, sex, and the first three genetic principal components (PCs), and the genomic inflation factor (λ) based on median *χ*^2^ was 1.00353, indicating negligible inflation and minimal population stratification. In the top-right corner, the quantile–quantile (QQ) plot compares the observed –log_10_(*p*-values) (*y*-axis) to the expected –log_10_(*p*-values) under the null hypothesis (*x*-axis), with the red diagonal line representing the expected null distribution and black dots representing the observed data; the close correspondence between the two indicates that the test statistics follow the expected null distribution, with deviation at the upper tail consistent with true associations rather than systematic bias. **b **Association of rs844607 genotype with the prevalence of nasolabial folds among participants with regular Sun exposure from the Singapore/Malaysia Cross-sectional Genetics Epidemiology Study (SMCGES). This figure presents a 100% stacked bar chart illustrating the proportional distribution of nasolabial fold cases and controls across the three genotypes of rs844607 (CC, TC, TT). The *x*-axis represents the genotype categories, while the *y*-axis indicates the percentage of participants (0 to 100%) within each genotype group. The major allele is C and the minor allele is t. An additive trend is observed, with the proportion of nasolabial fold cases increasing stepwise from 8% in CC homozygotes, to 18% in TC heterozygotes, and to 24% in TT homozygotes. The *χ*^2^ test for trend (Extended Mantel–Haenszel chi-square) demonstrates a significant dose-dependent relationship between the minor t allele and nasolabial folds (*χ*^2^ = 23.67, *p* < 0.001). Each of the case/control distributions in the three genotypes are significantly different from one another, with *χ*^2^ test *p*-values of 1.33 × 10^−5^ (CC vs TC), 1.16 × 10^−4^ (TC vs TT), and 1.44 × 10^−4^ (CC vs TT). The total sample size is *n* = 887, corresponding to genotyped participants with regular Sun exposure as defined in Fig. 1a. **c** Adjusted odds ratios (AORs) for nasolabial folds according to rs844607 genotypes among participants with regular Sun exposure from the Singapore/Malaysia Cross-sectional Genetics Epidemiology Study (SMCGES). This bar chart displays the adjusted odds ratios for nasolabial folds across the three genotypes of rs844607 (CC, TC, TT) in participants with regular Sun exposure (*n* = 887, as defined in Fig. 1a). The major allele is C and the minor allele is t. The genotype CC serves as the reference group (AOR = 1.00), indicated by a black dotted horizontal line at AOR = 1.00. Each bar represents the AOR with corresponding 95% confidence intervals (CIs) shown as solid black lines capped with vertical bars, and the point estimates are denoted by solid black circles. The adjusted *p*-value is displayed directly above each mean estimate. Compared with CC, individuals with TC exhibit a significantly higher odds of nasolabial folds with an AOR [95% CI] = 3.36 [2.12–5.39], *p*-value = 3.33 × 10^−7^, while those with TT show even greater odds: AOR [95% CI] = 4.87 [2.07–10.82], *p*-value = 1.56 × 10^−4^ after adjustment for age and sex. The case/control counts for each genotype are 42/487 for CC, 57/259 for TC, and 10/32 for TT, indicating a stepwise increase in odds with each additional copy of the minor t allele. Abbreviations: AOR, adjusted odds ratio; CI, confidence interval; SMCGES, Singapore/Malaysia Cross-sectional Genetics Epidemiology Study

The minor t allele of the lead SNP rs844607 was significantly associated with greater odds of nasolabial folds (AOR [95% CI] = 2.67 [1.89–3.77], *p* = 2.27 × 10^–8^). Moreover, a continuous signal drag exists across multiple SNPs within the *SAMD5* region, indicating gene-level associations rather than isolated SNPs (Fig. 1). As rs844607 is near the 3′ end of *SAMD5*, we investigated whether 3′-end SNP variants may influence *SAMD5* expression by altering miRNA binding dynamics.

To formally evaluate whether the association with lead SNP rs844607 differs by Sun exposure, we analysed the full dataset, including participants with both regular and non-regular Sun exposure, using a logistic regression model with a SNP × Sun exposure interaction term. In this model, we adjusted for age, sex, PC1, PC2, PC3, and Sun exposure. The minor t allele of rs844607 showed an interaction AOR of 2.986 (95% CI 1.961–4.547) with a *p*-value of 3.43 × 10^−7^. While this does not reach the conventional genome-wide significance threshold, it is consistent with the stratified analyses, supporting the conclusion that the effect of rs844607 on nasolabial folds is likely stronger among individuals with regular Sun exposure.

### SNP variants downregulate *SAMD5* expression by modulating miRNA binding

**Fig. 2:**
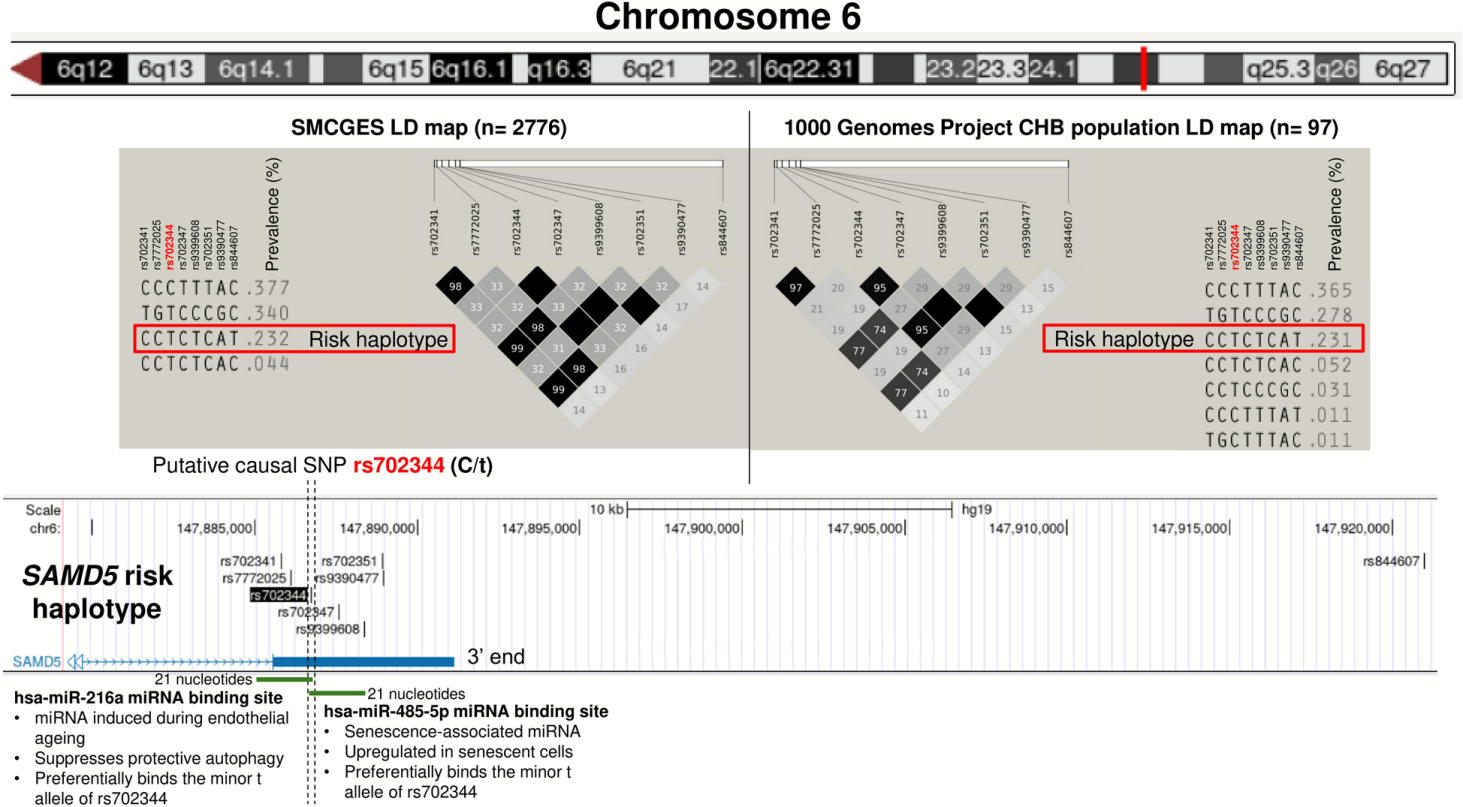
Linkage disequilibrium (LD) and functional annotation of the *SAMD5* locus on chromosome 6q24.3 associated with nasolabial folds in the Singapore/Malaysia Cross-sectional Genetics Epidemiology Study (SMCGES). The upper panel shows the q-arm of chromosome 6 from the UCSC Genome Browser (GRCh37/hg19), with the region of interest marked by a solid red vertical line at 6q24.3 corresponding to the 3’ end of the *SAMD5* gene (bottom panel). Gene coordinates were based on GENCODE V48lift37. Eight single nucleotide polymorphisms (SNPs): rs702341, rs7772025, rs702344, rs702347, rs9399608, rs702351, rs9390477, and rs844607, form an LD block encompassing the risk haplotype CCTCTCAT, boxed in red. This haplotype represents the C allele of rs702341, the C allele of rs7772025, the T allele of rs702344 (highlighted in red as the putative causal variant), the C allele of rs702347, the T allele of rs9399608, the C allele of rs702351, the A allele of rs9390477, and the T allele of rs844607. The prevalence of this risk haplotype is similar irrespective of population source: 23.2% in the SMCGES and 23.1% in the 1000 Genomes Project Han Chinese in Beijing (CHB) population. In SMCGES, when rs844607 carries the T allele, the rest of the haplotype always forms CCTCTCAT, indicating perfect LD; in CHB, the same configuration predominates, with the only alternative, CCCTTTAT, occurring in 1.1%. The LD heatmap shows pairwise *R*^2^ values (0–1.0) as a gradient from white (0.0) to black (1.0), with black squares lacking numbers indicating complete LD (*R*^2^ = 1.0). The bottom panel shows the *SAMD5* 3′ region with rs702344 boxed in black and flanked by black vertical dotted lines. The major allele is C and the minor allele is t. hsa-miR-216a, an endothelial ageing-induced miRNA that suppresses protective autophagy, is predicted to bind a 21-nucleotide region encompassing rs702344 and 20 upstream bases (AATTCTCCTGCCTCGGCCTCT). Binding is stronger for the minor t allele (miRANDA score = 151, ΔG = − 20.61 kcal/mol) than the C allele (score = 146, ΔG = − 18.81 kcal/mol). hsa-miR-485-5p, a senescence-associated miRNA upregulated in ageing cells, binds a 21-nucleotide region spanning rs702344 and 20 downstream bases, also showing stronger affinity for the minor t allele (score = 157, ΔG = − 22.36 kcal/mol) than the C allele (score = 152, ΔG = − 20.44 kcal/mol). These results indicate that the minor t allele of rs702344 enhances miRNA binding stability for both miRNAs, suggesting a miRNA-related mode of action linking *SAMD5* regulation to nasolabial fold development. Abbreviations: ΔG, Gibbs free energy; CHB, Han Chinese in Beijing; LD, linkage disequilibrium; miRNA, microRNA; *SAMD5*, sterile alpha motif domain containing 5; SMCGES, Singapore/Malaysia Cross-sectional Genetics Epidemiology Study; SNP, single nucleotide polymorphism; UCSC, University of California, Santa Cruz

Bioinformatic analyses using miRanda identified seven SNPs at the 3′ end of *SAMD5*, as potential binding sites for 21 micro RNAs (miRNA). They form a risk haplotype with the lead SNP rs844607. This haplotype is present in ~ 23% of individuals irrespective of population source (1kGP CHB population or SMCGES) (Fig. 2).

eQTL data from the Genotype-Tissue Expression (GTEx) portal showed that the minor t allele of rs844607 is associated with significantly decreased *SAMD5* expression across multiple tissue types (normalised effect size = − 0.72, *p* = 2.79 × 10^−52^), in an additive effect manner. Independent evidence from the eQTLGen consortium corroborated this finding (*Z* = 6.16, *p* = 6.59 × 10^−6^) and the SMCGES showed similar trends.

We therefore hypothesise that the association between the *SAMD5* risk haplotype and nasolabial folds is mediated by miRNA-dependent downregulation of gene expression. miRNAs typically suppress gene expression by binding to the 3′ untranslated region (3′ UTR) of target genes. Thus, SNP variants within the risk haplotype should enhance miRNA binding affinity and stability to repress *SAMD5* expression.

Ten of the 21 miRNAs showed concordant patterns, binding the risk haplotype favourably. Among the concordant miRNAs, miRNAs hsa-miR-216a and hsa-miR-485-5p are functionally relevant miRNAs requiring further investigation.

### The putative causal SNP rs702344 enhances the binding affinity of two biologically relevant miRNAs to the *SAMD5* gene

Both hsa-miR-216a and hsa-miR-485-5p are miRNAs upregulated with age. hsa-miR-216a suppresses protective autophagy and is elevated during endothelial ageing [22], while hsa-miR-485-5p is upregulated in replicatively senescent cells [23]. Their binding to *SAMD5* is influenced by the SNP rs702344 within the *SAMD5* risk haplotype, where the risk allele is t and the alternate allele is C.

For hsa-miR-216a, miRanda predicts binding to a 21-nucleotide sequence including rs702344 and the 20 upstream nucleotides. The t allele strengthens binding (alignment score = 151, ΔG = − 19.64 kcal/mol), whereas the C allele binds only to the 20 upstream nucleotides, resulting in weaker binding (score = 146, ΔG = − 18.81 kcal/mol). A higher alignment score and more negative ΔG indicate a stronger and more stable interaction.

Similarly, hsa-miR-485-5p binds to a 21-nucleotide stretch including rs702344 and the 20 downstream nucleotides. The binding is stronger with the t allele (score = 157, ΔG = − 22.36 kcal/mol) and weaker with the C allele (score = 152, ΔG = − 20.44 kcal/mol).

Approximately 23.1–23.2% of the population carry the risk haplotype with the minor t allele of rs702344, which enhances binding affinity and stability of both miRNAs to *SAMD5*, downregulating its expression and promoting an ageing-like phenotype through miRNA-mediated repression of a gene involved in maintaining skin homeostasis.

### Evidence linking *SAMD5*, gene expression, and nasolabial folds

Consistent with the above findings, a DEG analysis of National Center for Biotechnology Information (NCBI) Gene Expression Omnibus (GEO) dataset GSE200002 revealed that older individuals exhibit significantly lower *SAMD5* expression than younger counterparts (log fold change = − 0.639; adjusted *p* = 7.57 × 10^−3^). A similar age-related declining trend was independently observed in the SMCGES.

We further evaluated *SAMD5* expression in skin tissue using publicly available RNA sequencing data. Specifically, we analysed the Gene Expression Omnibus dataset GSE294121, which profiled gene expression in ultraviolet radiation (UVR)-induced photo-ageing in human dermal fibroblasts and assessed transcriptional changes following retinoid treatment. Retinoids, including retinoic acid and related derivatives, are well-established to produce both clinical and histological improvements in photo-ageing by restoring the extracellular matrix [24].

We examined the comparison between UVR-induced photo-aged control skin and retinoid-treated skin. *SAMD5* expression was significantly higher in retinoid-treated samples relative to untreated UVR-damaged controls (*p* = 3.00 × 10^−2^). Retinoid treatment is known to enhance extracellular matrix remodelling and promote recovery from photo-ageing-related damage. In our study, reduced *SAMD5* expression was associated with greater nasolabial fold severity, a photo-ageing phenotype. Consistent with this, in GSE294121, retinoid-treated samples exhibited significantly higher *SAMD5* expression compared with untreated UVR-damaged controls (adj. *p* = 3.00 × 10^–2^). The concordant direction of effect suggests that increased *SAMD5* expression may be linked to biological pathways associated with improved photo-ageing outcomes and less wrinkling.

Future analyses using publicly available human single-cell RNA-seq datasets of skin involving fibroblasts, keratinocytes, and other dermal cell populations, would further refine the cellular specificity of *SAMD5* expression and its role in skin ageing.

This finding aligns with GWAS results, showing that the minor t allele of the *SAMD5* risk haplotype increases susceptibility to nasolabial folds. Likewise, eQTL data from GTEx, eQTLGen, and SMCGES consistently demonstrate that the t allele reduces *SAMD5* expression in an additive manner.

**Fig. 3:**
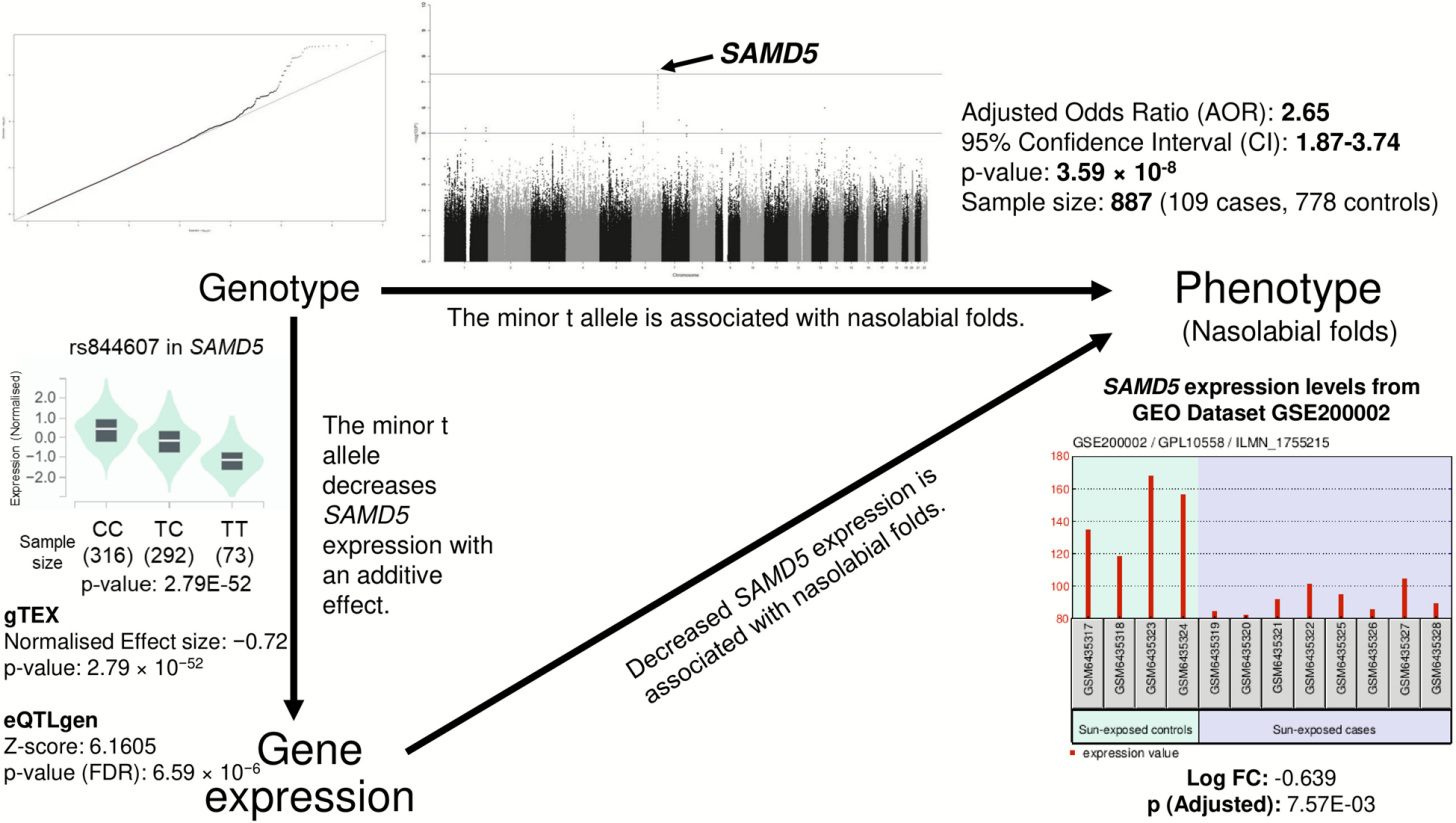
Mutually concordant genetic and expression-based evidence linking the minor t allele of rs844607 with nasolabial folds through *SAMD5* expression regulation. This figure summarises three complementary lines of evidence: genome-wide association study (GWAS), expression quantitative trait locus (eQTL), and differential gene expression (DEG), that together support the involvement of *SAMD5* in nasolabial folds. First, the GWAS evidence (previously described in Fig. 1a) shows a GWAS association between the minor t allele of rs844607 and nasolabial folds among participants with regular Sun exposure (*n* = 887), with the lead SNP reaching *p* = 2.27 × 10^−8^ and an adjusted odds ratio (AOR) [95% confidence interval (CI)] = 2.67 [1.89–3.77]. The Manhattan and quantile–quantile (QQ) plots indicate a clear association peak at the *SAMD5* locus and negligible genomic inflation (λ = 1.00353), suggesting a genuine signal rather than population stratification. Second, eQTL analyses from the Genotype-Tissue Expression (GTEx) portal and the eQTLGen consortium consistently show that the minor t allele of rs844607 decreases *SAMD5* expression in an additive manner. In GTEx, the violin plot of normalised expression shows progressively reduced *SAMD5* levels from CC to TC to TT genotypes (normalised effect size = − 0.72, *p* = 2.79 × 10^−52^; CC = 316, TC = 292, TT = 73), supporting the effect of varying copies of the minor t allele on *SAMD5* expression levels. Similarly, eQTLGen reports a significant negative association (*Z*-score = 6.1605, false discovery rate (FDR) *p*-value = 6.59 × 10^−6^). Third, differential expression analysis from the National Center for Biotechnology Information (NCBI) Gene Expression Omnibus (GEO) dataset GSE200002 demonstrates that among Sun-exposed individuals, chronologically older adults exhibit significantly reduced *SAMD5* expression (log fold change = − 0.639, adjusted *p* = 7.57 × 10^−3^). Collectively, these data form a coherent model in which the minor t allele of rs844607 enhances the binding affinity of hsa-miR-216a and hsa-miR-485-5p to *SAMD5*, resulting in reduced *SAMD5* transcription and diminished melanogenic potential. This reduction may weaken melanin-based UV protection, thereby accelerating photo-ageing processes such as nasolabial fold development. The mutually supported GWAS, eQTL, and DEG findings strengthens the conclusion that *SAMD5* genetic variants modulate gene expression to influence the skin’s photo-protective capacity and facial wrinkle formation in Sun-exposed individuals. Abbreviations: λ, Genomic inflation factor; AOR, adjusted odds ratio; CI, confidence interval; DEG, differentially expressed gene; eQTL, expression quantitative trait locus; FDR, false discovery rate; GEO, gene expression omnibus; GTEx, genotype-tissue expression portal; GWAS, genome-wide association study; NCBI, National Center for Biotechnology Information; *SAMD5*, sterile alpha motif domain containing 5

Although each dataset is associative, the concordant evidence across independent populations and methodologies supports a unified mechanism of action: the risk haplotype promotes nasolabial folds by downregulating *SAMD5* expression, likely through promoting the binding of miRNAs such as hsa-miR-216a and hsa-miR-485-5p (Fig. 3).

### *SAMD5* expression promotes melanogenesis

*SAMD5* plays a role in the regulation of melanogenesis by interacting with SMAD4 in the TGF-β signalling pathway [25]. This forms part of a downstream signalling cascade that activates MITF, a master transcriptional regulator of melanogenic genes [25].

Therefore, SAMD5 indirectly promotes the synthesis of melanin, through activating MITF. Melanin is a pigment with potent photoprotective and antioxidant properties. Melanin scavenges and quenches reactive oxygen species (ROS) generated by UVR, thereby limiting oxidative damage [26]. The extent of UV-induced injury varies from tanning to burning and peeling, with tanning representing minimal damage and sunburn characterised by epidermal peeling on the other extreme end as the body sheds UV-damaged keratinocytes [27].

Collectively, these observations suggest that *SAMD5* contributes to UV resilience by triggering melanogenesis via the TGF-β/SMAD4-MITF signalling pathway.

### Individuals with burning skin types are more likely to carry TC/TT genotypes

**Fig. 4:**
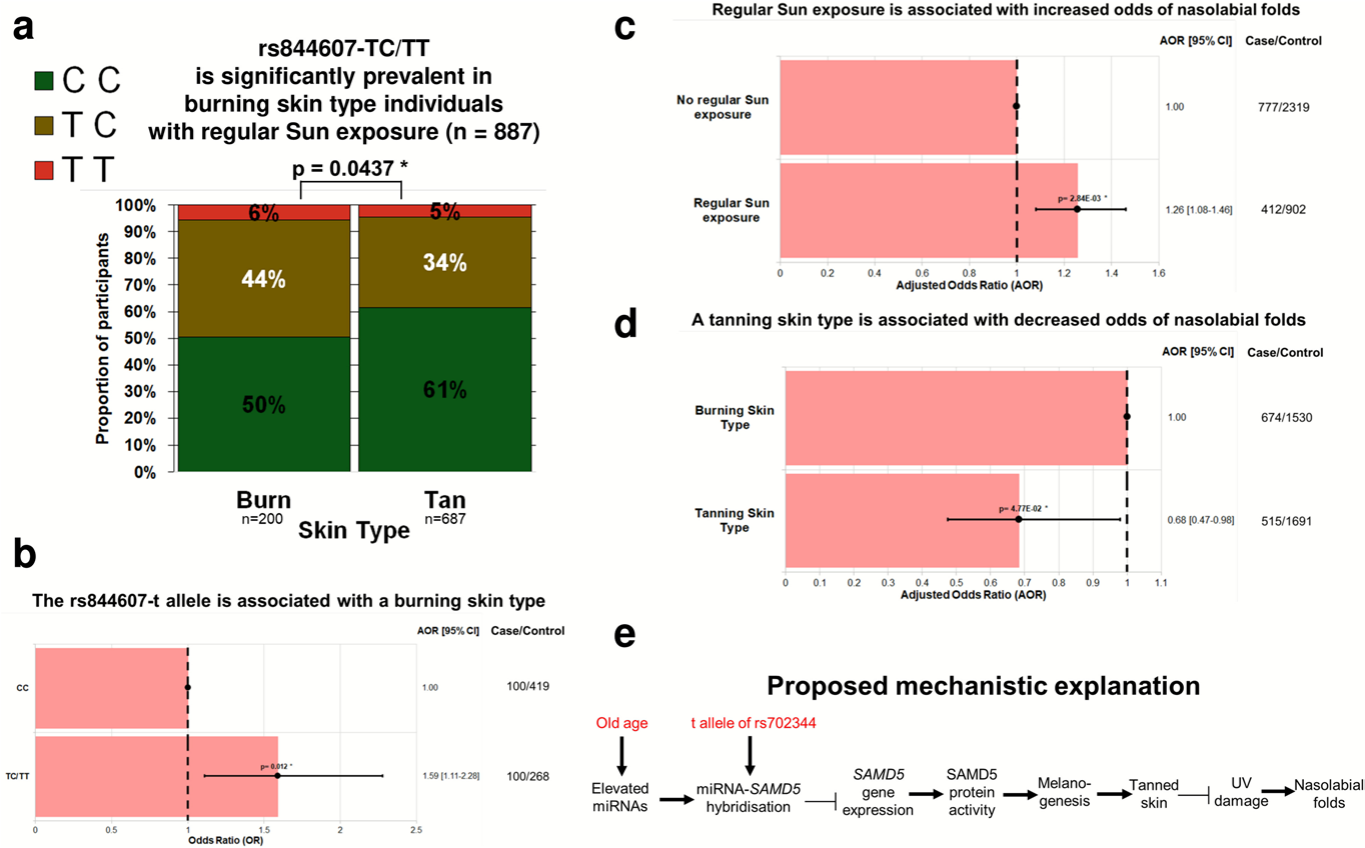
**a** A 100% stacked bar chart showing the percentage distribution of rs844607 genotypes (CC, TC, and TT) across two skin types: burning (*n* = 200) and tanning (*n* = 687). The *y*-axis represents percentage values from 0 to 100%. The genotype distributions differ significantly between the two skin types (*p* = 0.0437). Individuals carrying the TC and TT genotypes at rs844607 are significantly more prevalent among burning skin type individuals with regular Sun exposure. Sun exposure was quantified using a validated open-ended survey question on outdoor recreational activity frequency and duration by Morales-Sánchez *et al.* (2014), while skin type classification (burning or tanning) was based on the Fitzpatrick Skin Type using a validated questionnaire by Køster et al. (2017). **b** A bar chart showing adjusted odds ratios (AORs) for burning skin type across rs844607 genotypes (CC, TC, TT) among participants with regular Sun exposure from the Singapore/Malaysia Cross-sectional Genetics Epidemiology Study (SMCGES) (*n* = 887). Skin type classification (burning or tanning) was based on the Fitzpatrick Skin Type using a validated questionnaire by Køster et al*.* (2017). The major allele is C, and the minor allele is t. The genotype CC serves as the reference group (AOR = 1.00), represented by a black dotted horizontal line. Each bar depicts the AOR with 95% confidence intervals (CIs) shown as solid black lines capped with vertical bars, and point estimates marked by solid black circles. Adjusted *p*-values are displayed above the means. Compared with CC, individuals carrying at least one copy of the minor t allele have a significantly higher odds of exhibiting a burning skin type (AOR [95% CI] = 1.59 [1.11–2.28], *p*-value = 0.012), adjusted for age and sex. Overall, the minor t allele of rs844607 is associated with a burning skin type in regularly Sun-exposed individuals. Case/control counts are 100/419 for CC and 100/268 for TC and TT combined. Abbreviations: AOR, adjusted odds ratio; CI, confidence interval; SMCGES, Singapore/Malaysia Cross-sectional Genetics Epidemiology Study. **c **A bar chart showing adjusted odds ratios (AORs) for nasolabial folds according to regular Sun exposure among participants from the Singapore/Malaysia Cross-sectional Genetics Epidemiology Study (SMCGES) (*n* = 4410). The chart compares two categories: no regular Sun exposure (reference group, AOR = 1.00) and regular Sun exposure. Sun exposure was quantified using a validated open-ended survey on outdoor recreational activity frequency and duration by Morales-Sánchez *et al.* (2014). AOR = 1.00 is indicated by a black dotted horizontal line. Each bar depicts the AOR with 95% confidence intervals (CIs) shown as solid black lines capped with vertical bars, and point estimates marked by solid black circles, with adjusted *p*-values displayed above each mean. Compared with the reference group, individuals with regular Sun exposure have a significantly higher odds of nasolabial folds (AOR [95% CI] = 1.26 [1.08–1.46], *p* = 2.84 × 10^−3^), after adjustment for age and sex. Case/control counts are 777/2319 for no regular Sun exposure and 412/902 for regular Sun exposure. Abbreviations: AOR, adjusted odds ratio; CI, confidence interval; SMCGES, Singapore/Malaysia Cross-sectional Genetics Epidemiology Study. **d** A bar chart showing adjusted odds ratios (AORs) for nasolabial folds according to skin type among participants from the Singapore/Malaysia Cross-sectional Genetics Epidemiology Study (SMCGES) (*n* = 4410). The chart compares two categories: melanin-poor burning skin type (reference group, AOR = 1.00) and melanin-rich tanning skin type. The reference group is indicated by a black dotted horizontal line at AOR = 1.00. Skin type classification was based on the Fitzpatrick Skin Type using a validated questionnaire by Køster et al*.* (2017). Each bar depicts the AOR with 95% confidence intervals (CIs) shown as solid black lines capped with vertical bars, and point estimates marked by solid black circles, with adjusted *p*-values displayed above each mean. Compared with the reference group, individuals with tanning skin type have a significantly lower odds of nasolabial folds (AOR [95% CI] = 0.68 [0.47–0.98], *p* = 4.77 × 10^−2^), after adjustment for age and sex. Case/control counts are 674/1530 for burning skin type and 515/1691 for tanning skin type. Abbreviations: AOR, adjusted odds ratio; CI, confidence interval; SMCGES, Singapore/Malaysia Cross-sectional Genetics Epidemiology Study. **e** Proposed mechanistic model linking chronological ageing, rs702344 genotype, and nasolabial folds. Chronological ageing induces the upregulation of microRNAs, notably hsa-miR-216a, an endothelial ageing-associated miRNA that suppresses protective autophagy, and hsa-miR-485-5p, a senescence-associated miRNA upregulated in senescent cells. Both miRNAs hybridise to the 3′ end of the *SAMD5* transcript, with transcripts carrying the minor t allele at rs702344 promoting stronger and more stable miRNA binding. This enhanced miRNA-*SAMD5* interaction reduces *SAMD5* gene expression and protein activity. Elevated *SAMD5* expression normally activates the TGF-β/SMAD4-MITF pathway, enhancing melanogenesis and melanin-mediated photoprotection. Consequently, individuals with tanning skin types, naturally higher in melanin, maintain better UV defence and reduced risk of nasolabial fold formation. The presence of the minor t allele at rs702344 recapitulates the effects of ageing by downregulating *SAMD5* via miRNA binding, so that individuals carrying this allele who are exposed to regular Sun exposure experience an accelerated ageing phenotype, manifesting as early-onset nasolabial folds. Abbreviations: miRNA, microRNA; MITF, microphthalmia-associated transcription factor; *SAMD5*, sterile alpha motif domain containing 5; TGF-β, transforming growth factor beta

Individuals with burning skin types, as classified by the Fitzpatrick Scale, have lower levels of photo-protective melanin, making them more susceptible to UV damage. Among the SMCGES, 50% of burning skin type individuals carried at least one t allele at rs702344, compared with 39% of tanning skin type individuals (*p* = 0.0437). Logistic regression showed that carrying at least one t allele increased the odds of a burning skin type by 1.6-fold (95% CI, 1.11–2.28; *p* = 0.012) after adjusting for age and sex (Fig. 4). These findings suggest that *SAMD5* genetic variation influences individual UV sensitivity, likely via effects on melanin production.

### The protective effect of *SAMD5* expression is modulated by skin type

Sustained Sun exposure is associated with increased nasolabial folds, with the SMCGES showing 1.26-fold higher odds (95% CI, 1.08–1.46; *p* = 2.8 × 10^−3^) after adjusting for age and sex (Fig. 4). Elevated *SAMD5* expression appears protective, activating the TGF-β/SMAD4-MITF pathway to enhance melanogenesis and melanin-mediated photo-protection. Consistently, individuals with tanning skin types (i.e., naturally higher in melanin) have reduced odds of nasolabial folds (AOR = 0.68; 95% CI, 0.47–0.98; *p* = 0.048) compared to burning skin types (Fig. 4).

Conversely, ageing and the t allele of rs702344 undermine this protection. Ageing elevates two miRNAs of interest: the t allele strengthens binding of hsa-miR-216a and hsa-miR-485-5p, downregulating *SAMD5*. Reduced *SAMD5* impairs melanin production and UV protection, accelerating nasolabial fold formation and recapitulating an ageing phenotype.

## Discussion

### *SAMD5* contributes evidence linking pigmentation pathways to facial wrinkle development

Our study identifies *SAMD5* as a novel gene associated with the development of nasolabial folds in Sun-exposed individuals. While *SAMD5* has previously been implicated in melanogenesis, this is the first evidence linking it to a facial wrinkling phenotype. Our findings are supported by concordant genetic and expression-based data. These data suggest a coherent model in which *SAMD5* interacts with Sun exposure to modulate nasolabial fold development, potentially via SNPs that influence the binding strength and stability of miRNAs biologically relevant to the ageing process.

The association of *SAMD5* with nasolabial folds adds to growing evidence that genes involved in melanogenesis (e.g., *MC1R*, *IRF4*, *SLC45A2*) contribute not only to skin pigmentation but also to structural ageing. Indeed, wrinkling phenotypes including solar elastosis, wrinkles under the eyes, glabellar frown lines, Crow's Feet wrinkles, and forehead wrinkles have already been linked to melanogenesis-related genes [28, 29].

### *SAMD5* as a newly discovered contributor to nasolabial folds

Genome-wide analysis identified *SAMD5* variants, including rs844607 and rs702344, as significantly associated with nasolabial folds, but only in individuals with regular Sun exposure. This Sun-specific effect highlights the influence of UV exposure on the manifestation of skin ageing phenotypes and may explain why these associations were previously overlooked.

### Mechanistic insights: miRNA-mediated downregulation of *SAMD5*

Bioinformatic analyses suggest that the t allele of rs702344 at the 3′ end of *SAMD5* enhances binding of two miRNAs (hsa-miR-216a and hsa-miR-485-5p), which are upregulated with chronological age. This increased binding downregulates *SAMD5* expression, as supported by eQTL data from GTEx, eQTLgen, and the SMCGES, providing a plausible mechanism linking the minor t allele of rs702344 to increased nasolabial fold formation.

### Elevated *SAMD5* expression leads to melanogenesis and protective odds against nasolabial folds

SAMD5 interacts with SMAD4 in the TGF-β pathway to promote MITF expression and melanogenesis, providing natural protection against UV-induced oxidative damage. Reduced *SAMD5* expression, as seen with the minor t allele of rs702344, weakens this photo-protective effect, accelerating the development of nasolabial folds in Sun-exposed individuals. This is consistent with observations that tanning skin types, with higher melanin, are less prone to nasolabial folds (AOR = 0.68). supporting the continued use of skin type as a parameter in skin ageing research.

Thus, the minor t allele of rs702344 appears to functionally recapitulate a wrinkling phenotype that would have otherwise appeared at a chronologically later time point. Having the minor t allele diminishes the protective effects conferred by *SAMD5*-mediated melanogenesis in Sun-exposed skin.

### Coherent model on the impact of *SAMD5* and Sun exposure on nasolabial folds

We present a three-way coherent association model linking genotype, gene expression, and phenotype. The minor t allele of rs702344 enhances binding of two miRNAs to *SAMD5*, reducing its expression and melanogenic potential. This weakens melanin-mediated UV defence, leading to early-onset nasolabial folds. Evidence from GWAS, eQTL, and DEG analyses mutually strengthen and supports our hypothesis that *SAMD5* variants modulate gene expression to influence the skin’s photo-protective capacity and wrinkle development in Sun-exposed individuals.

### Implications and future directions

The discovery of *SAMD5* as a novel gene linked to facial wrinkling provides a foundation for exploring therapeutic strategies to mitigate skin ageing. Future research should investigate modulation of *SAMD5* or its interacting miRNAs, validate their functional roles, and assess the broader impact on pigmentation-related disorders and early-onset skin ageing.

The identification of *SAMD5* as a novel wrinkling gene opens new opportunities for understanding and exploring therapeutic strategies to mitigate facial ageing. Functional assays should be conducted in the future to validate the model and verify if either or both miRNAs are additively or synergistically responsible for *SAMD5* downregulation.

### Generalisability of our findings across populations

We previously reported in our systematic review that skin ageing phenotypes differ across populations by race, sex, and chronological age [2]. Comparative studies consistently report that Caucasian skin, which has relatively lower melanin content, is more susceptible to photo-damage and tends to exhibit earlier and more pronounced wrinkling and sagging [30]. In contrast, Asian skin, including Chinese and Japanese populations, generally has higher melanin content, which confers greater photo-protection, but aged Asian skin more frequently manifests pigmentary changes, such as hyperpigmented spots and uneven pigmentation [31]. For example, middle-aged Asians have been reported to develop pigment spots on the cheeks more often than age-matched Germans, whereas Germans more commonly exhibit wrinkles under the eyes [32]. Moreover, wrinkle morphology also differs, with Asians tending to display coarser and deeper wrinkles in specific regions, such as the forehead, perioral area, and the corners of the eyes (i.e., Crow's Feet wrinkles), whereas Caucasians often show relatively finer wrinkles on the cheeks and periocular region [33]. Data on other populations, including individuals of Hispanic or African ancestry, remain comparatively limited, although abnormal pigmentation appears more frequent in darker skin types relative to the Caucasians [34–36].

These well documented ethnic differences in the clinical presentation of skin ageing suggest that both environmental exposures, particularly UVR, and underlying genetic architecture may interact differently across populations. Given that our study was conducted in young ethnic Chinese adults, the identified interaction between *SAMD5* variants and regular Sun exposure on nasolabial fold development should be interpreted within this population context. Differences in baseline melanin content, photo-damage susceptibility, lifestyle, dietary habits, and living environment may modulate both the magnitude and the direction of gene–environment interactions in other racial groups [37].

While SMCGES participants of various ethnicities were recruited, the ethnic Chinese subgroup remains the only group with sufficient sample size for adequately-powered genome-wide interaction analyses [16–19]. As such, the present findings are currently most directly applicable to ethnic Chinese individuals. Future recruitment efforts to expand the representation of Malays, Indians, and other ethnic groups within the cohort will enable comparisons to be made to determine whether the interaction between *SAMD5* and Sun exposure is shared across populations, or whether population-specific genetic or environmental modifiers influence nasolabial fold development.

### Study limitations

In this study, Sun exposure was assessed using a self-reported questionnaire and is therefore susceptible to recall bias. Future investigations could strengthen exposure assessment by incorporating more objective measures, such as personal ultraviolet dosimetry.

Our identification of an association between *SAMD5* and nasolabial folds among individuals with regular Sun exposure was based on 109 cases. Although this sample size was sufficient to achieve genome-wide significance, it remains modest. Replication in larger and independent cohorts will be able to confirm the robustness and generalisability of this finding.

In addition, we proposed a miRNA-mediated regulatory model linking *SAMD5*, Sun exposure, and nasolabial fold development. This model is supported by bioinformatic predictions and genetic association evidence. However, direct functional validation is required to establish causality. Experimental approaches such as luciferase reporter assays, as well as knockdown or overexpression studies in relevant skin cell models, would provide more definitive evidence for the proposed regulatory mechanism.

## Anthropological perspective on how genetics and Sun exposure shape early ageing patterns

From a physiological anthropological perspective, these findings show how the ageing face reflects a continuous interaction between genetics, everyday Sun exposure, and local and cultural habits among young ethnic Chinese adults in Singapore and Malaysia. In this tropical setting, UV exposure is a constant physiological stressor, and variations in *SAMD5*, particularly the regulatory effects associated with rs702344, appear to influence how effectively individuals produce melanin and maintain UV resilience. Those carrying alleles that reduce *SAMD5* expression may generate less photo-protective pigmentation, leaving the skin more vulnerable to early-onset nasolabial folds. This highlights how differences in gene regulation can shape visible features early in adulthood.

These biological patterns are closely tied to the social and behavioural routines through which people move in their daily lives. Time spent outdoors, occupational exposure, and cultural preferences surrounding skin tone all affect how UV interacts with underlying pigmentation pathways. The observed links between skin type, Sun-exposure habits, and nasolabial folds indicate that skin ageing arises from complex interactions between lived experience and genetics. In the SMCGES, the combination of strong tropical UV, inherited genetic variations in *SAMD5*, and different Sun-related behaviours leads to the emergence of nasolabial folds. These results emphasise the importance of understanding skin ageing as a process shaped continuously by biological regulation, environmental conditions, and cultural practice.

## Conclusion

In conclusion, we report a previously unrecognised association between *SAMD5* and nasolabial folds, mediated by miRNA-dependent gene regulation and modulated by Sun exposure and skin type. Our finding highlights *SAMD5* as a novel target gene for developing anti-wrinkling strategies and exemplifies how integrating genomics with transcriptomics and environmental factors can illuminate complex skin ageing mechanisms.

## Supplementary Information

Additional file 1: Genome-wide association study (GWAS) of nasolabial folds in participants with regular Sun exposure from the Singapore/Malaysia Cross-sectional Genetics Epidemiology Study (SMCGES). This Manhattan plot, generated using the qqman package on R with the RStudio interface in RStudio version 4.3.1, shows the genome-wide distribution of single nucleotide polymorphism (SNP) associations with nasolabial folds among all genotyped participants, regardless of Sun exposure status (*n* = 2776; cases = 344, controls = 2432). Each point represents an SNP, with chromosomal position on the x-axis and the corresponding –log_10_(*p*-value) on the y-axis. The blue horizontal line denotes the suggestive significance threshold (*p* = 1 × 10^–5^), while the red line marks the genome-wide significance threshold (*p* = 5 × 10^–8^). A clear drag of association signals is observed on chromosome 6 near the *SAMD5* locus, with the lead SNP rs844607 exhibiting the strongest association; the minor t allele is associated with nasolabial folds, with an adjusted odds ratio (AOR) [95% confidence interval (CI)] = 1.21 [1.00–1.47], *p* = 0.05. The GWAS included 3,565,291 SNP variants genotyped from buccal cell DNA of 2,776 Chinese participants. The analysis was adjusted for age, sex, and the first three genetic principal components (PCs). In the top-right corner, the quantile–quantile (QQ) plot compares the observed –log_10_(*p*-values) (y-axis) to the expected –log_10_(*p*-values) under the null hypothesis (x-axis), with the red diagonal line representing the expected null distribution and black dots representing the observed data; the close correspondence between the two indicates that the test statistics follow the expected null distribution, with deviation at the upper tail consistent with true associations rather than systematic bias.

Additional file 2: Association of rs844607 genotype with the prevalence of nasolabial folds among all genotyped participants from the Singapore/Malaysia Cross-sectional Genetics Epidemiology Study (SMCGES) regardless of Sun exposure status. This figure presents a 100% stacked bar chart illustrating the proportional distribution of nasolabial fold cases and controls across the three genotypes of rs844607 (CC, TC, TT). The x-axis represents the genotype categories, while the y-axis indicates the percentage of participants (0 to 100%) within each genotype group. The major allele is C and the minor allele is t. An additive trend is observed, with the proportion of nasolabial fold cases increasing stepwise from 11% in CC homozygotes, to 14% in TC heterozygotes, and to 14% in TT homozygotes. The χ^2^ test for trend (Extended Mantel–Haenszel Chi-square) demonstrates a significant dose-dependent relationship between the minor t allele and nasolabial folds (χ^2^ = 4.25, *p* = 0.0392). The χ^2^ test *p*-values for the case/control distributions in the three genotypes are 0.03 (CC vs TC), 0.09 (TC vs TT), and 0.12 (CC vs TT). The total sample size is *n* = 2776. Abbreviations: χ^2^ test, chi-square test; SMCGES, Singapore/Malaysia Cross-sectional Genetics Epidemiology Study.

Additional file 3: Adjusted odds ratios (AORs) for nasolabial folds according to rs844607 genotypes among all genotyped participants from the Singapore/Malaysia Cross-sectional Genetics Epidemiology Study (SMCGES) regardless of Sun exposure status. This bar chart displays the adjusted odds ratios for nasolabial folds across the three genotypes of rs844607 (CC, TC, TT) in all genotyped participants (*n* = 2776). The major allele is C and the minor allele is t. The genotype CC serves as the reference group (AOR = 1.00), indicated by a black dotted horizontal line at AOR = 1.00. Each bar represents the AOR with corresponding 95% confidence intervals (CIs) shown as solid black lines capped with vertical bars, and the point estimates are denoted by solid black circles. The adjusted *p*-value is displayed directly above each mean estimate. Compared with CC, individuals with TC exhibit a significantly higher odds of nasolabial folds with an AOR [95% CI] = 1.32 [1.03–1.70], *p*-value = 0.029. Compared with CC, individuals with TT exhibit a higher odds of nasolabial folds with an AOR [95% CI] = 1.24 [0.72–2.04], *p*-value = 0.408 after adjustment for age and sex. The case/control counts for each genotype are 187/1474 for CC, 135/824 for TC, and 22/134 for TT, indicating a stepwise increase in odds with each additional copy of the minor t allele. Abbreviations: AOR, adjusted odds ratio; CI, confidence interval; SMCGES, Singapore/Malaysia Cross-sectional Genetics Epidemiology Study.

Additional file 4: Genome-wide association study (GWAS) of nasolabial folds in participants with regular Sun exposure from the Singapore/Malaysia Cross-sectional Genetics Epidemiology Study (SMCGES). This Manhattan plot, generated using the qqman package on R with the RStudio interface in RStudio version 4.3.1, shows the genome-wide distribution of single nucleotide polymorphism (SNP) associations with nasolabial folds among participants reporting no regular Sun exposure (*n* = 1889; cases = 235, controls = 1654). Each point represents an SNP, with chromosomal position on the x-axis and the corresponding –log_10_(*p*-value) on the y-axis. The blue horizontal line denotes the suggestive significance threshold (*p* = 1 × 10^–5^), while the red line marks the genome-wide significance threshold (*p* = 5 × 10^–8^). A clear drag of association signals is observed on chromosome 6 near the *SAMD5* locus, with the lead SNP rs844607 exhibiting the strongest association; the minor t allele is associated with nasolabial folds, with an adjusted odds ratio (AOR) [95% confidence interval (CI)] = 0.83 [0.65–1.07], *p* = 0.15. The GWAS included 3,565,291 SNP variants genotyped from buccal cell DNA of 1,889 Chinese participants. The analysis was adjusted for age, sex, and the first three genetic principal components (PCs). In the top-right corner, the quantile–quantile (QQ) plot compares the observed –log_10_(*p*-values) (y-axis) to the expected –log_10_(*p*-values) under the null hypothesis (x-axis), with the red diagonal line representing the expected null distribution and black dots representing the observed data; the close correspondence between the two indicates that the test statistics follow the expected null distribution, with deviation at the upper tail consistent with true associations rather than systematic bias.

Additional file 5: Association of rs844607 genotype with the prevalence of nasolabial folds among participants with no regular Sun exposure from the Singapore/Malaysia Cross-sectional Genetics Epidemiology Study (SMCGES). This figure presents a 100% stacked bar chart illustrating the proportional distribution of nasolabial fold cases and controls across the three genotypes of rs844607 (CC, TC, TT). The x-axis represents the genotype categories, while the y-axis indicates the percentage of participants (0 to 100%) within each genotype group. The major allele is C and the minor allele is t. No trend is observed. The proportion of nasolabial fold cases is 13% in CC homozygotes, 12% in TC heterozygotes, and to 11% in TT homozygotes. The χ^2^ test for trend (Extended Mantel–Haenszel Chi-square) demonstrates no significant dose-dependent relationship between the minor t allele and nasolabial folds (χ^2^ = 0.532, *p* = 0.466). The χ^2^ test *p*-values for the case/control distributions in the three genotypes are 0.66 (CC vs TC), 0.51 (TC vs TT), and 0.47 (CC vs TT). The total sample size is *n* = 1889. Abbreviations: χ^2^ test, chi-square test; SMCGES, Singapore/Malaysia Cross-sectional Genetics Epidemiology Study.

Additional file 6: Adjusted odds ratios (AORs) for nasolabial folds according to rs844607 genotypes among participants with no regular Sun exposure from the Singapore/Malaysia Cross-sectional Genetics Epidemiology Study (SMCGES). This bar chart displays the adjusted odds ratios for nasolabial folds across the three genotypes of rs844607 (CC, TC, TT) in all genotyped participants (*n* = 1889). The major allele is C and the minor allele is t. The genotype CC serves as the reference group (AOR = 1.00), indicated by a black dotted horizontal line at AOR = 1.00. Each bar represents the AOR with corresponding 95% confidence intervals (CIs) shown as solid black lines capped with vertical bars, and the point estimates are denoted by solid black circles. The adjusted *p*-value is displayed directly above each mean estimate. Compared with CC, individuals with TC exhibit a lower odds of nasolabial folds with an AOR [95% CI] = 0.87 [0.64–1.19], *p*-value = 0.397. Compared with CC, individuals with TT exhibit a lower odds of nasolabial folds with an AOR [95% CI] = 0.64 [0.31–1.23], *p*-value = 0.211 after adjustment for age and sex. The case/control counts for each genotype are 145/987 for CC, 78/565 for TC, and 12/102 for TT, indicating a stepwise increase in odds with each additional copy of the minor t allele. Abbreviations: AOR, adjusted odds ratio; CI, confidence interval; SMCGES, Singapore/Malaysia Cross-sectional Genetics Epidemiology Study.

## Data Availability

All data generated or analysed during this study are included in this published article and its supplementary information files. The datasets used and/or analysed during the current study are available from the corresponding author (F. T. C.) on reasonable request.

## Abbreviations

1kGP: 1000 Genomes Project
3’ UTR: 3′ Untranslated region
AOR: Adjusted odds ratio
CHB population: Han Chinese in Beijing population
CI: Confidence interval
DEG: Differentially expressed genes
eQTL: Expression quantitative trait loci
eQTLgen consortium: Expression quantitative trait loci genomics consortium
FPKM: Fragments per kb of transcript per million mapped reads
gDNA: Genomic DNA
GEO dataset: Gene expression omnibus dataset
GTex portal: Genotype-tissue expression portal
GWAS: Genome-wide association study
HDB flat: Singapore’s Housing Development Board flat
HWE: Hardy-Weinberg equilibrium
INT: Inverse normal transformation
LD: Linkage disequilibrium
mRNA: Messenger RNA
miRNA: MicroRNA
NCBI: National Center for Biotechnology Information
NGS: Next-generation sequencing
NUS: National University of Singapore
OR: Odds ratio
PBMC: Peripheral blood mononuclear cell
PCA: Principal component analysis
PC1: First principal component
QQ plot: Quantile-quantile plot
ROS: Reactive oxygen species
RM: Malaysian ringgit
SAMD5: Sterile alpha motif domain-containing protein 5
SGD: Singapore dollars
SU: Sunway University
SMCGES: Singapore/Malaysia Cross-sectional Genetics Epidemiology Study
TPM: Transcripts per million
UTAR: University of Tunku Abdul Rahman
UVR: Ultraviolet radiation

## References

[1] Wong QYA, Chew FT. Defining skin aging and its risk factors: a systematic review and meta-analysis. Sci Rep. 2021;11(1):1–13. 10.1038/s41598-021-01573-z. 34764376 10.1038/s41598-021-01573-z PMC8586245

[2] Ng JY, Chew FT. A systematic review of skin ageing genes : gene pleiotropy and genes on the chromosomal band 16q24.3 may drive skin ageing. Sci Rep. 2022;12(1):1–23. 10.1038/s41598-022-17443-1. 35907981 10.1038/s41598-022-17443-1 PMC9338925

[3] Krutmann J, Bouloc A, Sore G, Bernard BA, Passeron T. The skin aging exposome. J Dermatol Sci. 2017;85(3):152–61. 10.1016/j.jdermsci.2016.09.015. 27720464 10.1016/j.jdermsci.2016.09.015

[4] Wong C, Ng JY, Sio YY, Chew FT. Genetic determinants of skin ageing: a systematic review and meta-analysis of genome-wide association studies and candidate genes. J Physiol Anthropol. 2025;44(1):1–17. 10.1186/s40101-025-00384-9. 39923055 10.1186/s40101-025-00384-9 PMC11806588

[5] Swift A, Liew S, Weinkle S, Garcia JK, Silberberg MB. The facial aging process from the “inside out.” Aesthet Surg J. 2021;41(10):1107–19. 10.1093/asj/sjaa339. 33325497 10.1093/asj/sjaa339 PMC8438644

[6] Ng JY, Wong QYA, Lim JJ, et al. A broad assessment of forty-one skin phenotypes reveals complex dimensions of skin ageing. J Physiol Anthropol. 2025;44(1):1–17. 10.1186/s40101-024-00383-2. 39923103 10.1186/s40101-024-00383-2 PMC11806859

[7] Lim JJ, Lim YYE, Ng JY, et al. An update on the prevalence, chronicity, and severity of atopic dermatitis and the associated epidemiological risk factors in the Singapore/Malaysia Chinese young adult population: a detailed description of the Singapore/Malaysia Cross-Sectional Genetics. World Allergy Organ J. 2022;15(12):100722. 10.1016/j.waojou.2022.100722. 36438192 10.1016/j.waojou.2022.100722 PMC9678778

[8] Wong QYA, Lim JJ, Ng JY, et al. Allergic rhinitis in Chinese young adults from the Singapore/Malaysia cross-sectional genetics epidemiology study (SMCGES) cohort: prevalence, patterns, and epidemiology of allergic rhinitis. World Allergy Organ J. 2022;15(10):100704. 10.1016/j.waojou.2022.100704. 36267097 10.1016/j.waojou.2022.100704 PMC9554817

[9] Wong QYA, Lim JJ, Ng JY, et al. An updated prevalence of asthma, its phenotypes, and the identification of the potential asthma risk factors among young Chinese adults recruited in Singapore. World Allergy Organ J. 2023;16(3):100757. 10.1016/j.waojou.2023.100757. 36968625 10.1016/j.waojou.2023.100757 PMC10033744

[10] Teo WY, Lim YYE, Sio YY, Say YH, Reginald K, Chew FT. Atopic dermatitis-associated genetic variants regulate LOC100294145 expression implicating interleukin-27 production and type 1 interferon signaling. World Allergy Organ J. 2024;17(2):100869. 10.1016/j.waojou.2023.100869. 38298829 10.1016/j.waojou.2023.100869 PMC10827559

[11] Sio YY, Gan WL, Ng WS, et al. The ERBB2 exonic variant Pro1170Ala modulates mitogen-activated protein kinase signaling cascades and associates with allergic asthma. Int Arch Allergy Immunol. 2023;184(10):1010–21. 10.1159/000530960. 37336194 10.1159/000530960

[12] Sio YY, Matta SA, Ng YT, Chew FT. Epistasis between phenylethanolamine N-methyltransferase and β2-adrenergic receptor influences extracellular epinephrine level and associates with the susceptibility to allergic asthma. Clin Exp Allergy. 2020;50(3):352–63. 10.1111/cea.13552. 31855300 10.1111/cea.13552

[13] Sio YY, Shi P, Matta SA, et al. Functional polymorphisms of the arachidonic acid pathway associate with risks and clinical outcomes of allergic diseases. Int Arch Allergy Immunol. 2023;184(6):609–23. 10.1159/000530393. 37231900 10.1159/000530393

[14] Sio YY, Shi P, Say YH, Chew FT. Functional variants in the chromosome 4q21 locus contribute to allergic rhinitis risk by modulating the expression of N-acylethanolamine acid amidase. Clin Exp Allergy. 2022;52(1):127–36. 10.1111/cea.13883. 33866639 10.1111/cea.13883

[15] Narins RS, Carruthers J, Flynn TC, et al. Validated assessment scales for the lower face. Dermatol Surg. 2012;38(2ptII):333–42. 10.1111/j.1524-4725.2011.02247.x. 22316189 10.1111/j.1524-4725.2011.02247.x

[16] Ng JY, Chew FT. Comparisons between eyebags, droopy eyelids, and eyebrow positioning identified by photo-numeric scales or identified by written descriptive scales: insights from the Singapore/Malaysia cross-sectional genetics epidemiology study (SMCGES) cohort. Skin Res Technol. 2024;30(2):1–12. 10.1111/srt.13620. 10.1111/srt.13620 PMC10878178 38376131

[17] Ng JY, Chew FT. Comparisons between Caucasian-validated photo-numeric scales and Korean-validated photo-numeric scales for photo-ageing. Insights from the Singapore/Malaysia cross-sectional genetics epidemiology study (SMCGES) cohort. Ski Res Technol. 2024;30(2):1–11. 10.1111/srt.13637. 10.1111/srt.13637 PMC11116842 38783624

[18] Ng JY, Zhou H, Li T, Chew FT. Comparisons between Caucasian- validated and Chinese-validated photo-numeric scales for assessing facial wrinkles. Sci Rep. 2024;14(1):1–12. 10.1038/s41598-024-78945-8. 39550380 10.1038/s41598-024-78945-8 PMC11569137

[19] Ng JY, Zhou H, Li T, Chew FT. Comparisons between wrinkles and photo-ageing detected and self-reported by the participant or identified by trained assessors reveal insights from Chinese individuals in the Singapore/Malaysia Cross-sectional Genetics Epidemiology Study (SMCGES) cohort. J Physiol Anthropol. 2024;43(1):1–22. 10.1186/s40101-024-00361-8. 38762735 10.1186/s40101-024-00361-8 PMC11102249

[20] Morales-Sánchez MA, Peralta-Pedrero ML, Domínguez-Gómez MA. Validation of a questionnaire to quantify the risk for skin cancer. Gac Med Mex. 2014;150(5):409–19. 25275843

[21] Køster B, Søndergaard J, Nielsen JB, Allen M, Olsen A, Bentzen J. The validated sun exposure questionnaire: association of objective and subjective measures of sun exposure in a Danish population-based sample. Br J Dermatol. 2017;176(2):446–56. 10.1111/bjd.14861. 27412948 10.1111/bjd.14861

[22] Menghini R, Casagrande V, Marino A, et al. MiR-216a: A link between endothelial dysfunction and autophagy. Cell Death Dis. 2014;5(1):1–9. 10.1038/cddis.2013.556. 10.1038/cddis.2013.556 PMC4040670 24481443

[23] Faraonio R, Salerno P, Passaro F, et al. A set of miRNAs participates in the cellular senescence program in human diploid fibroblasts. Cell Death Differ. 2012;19(4):713–21. 10.1038/cdd.2011.143. 22052189 10.1038/cdd.2011.143 PMC3307984

[24] Mukherjee S, Date A, Patravale V, Korting HC, Roeder A, Weindl G. Retinoids in the treatment of skin aging: an overview of clinical efficacy and safety. Clin Interv Aging. 2006;1(4):327–48. 10.2147/ciia.2006.1.4.327. 18046911 10.2147/ciia.2006.1.4.327 PMC2699641

[25] Maeda S, Hayashi M, Komiya S, Imamura T, Miyazono K. Endogenous TGF-β signaling suppresses maturation of osteoblastic mesenchymal cells. EMBO J. 2004;23(3):552–63. 10.1038/sj.emboj.7600067. 14749725 10.1038/sj.emboj.7600067 PMC1271802

[26] Tada M, Kohno M, Niwano Y. Scavenging or quenching effect of melanin on superoxide anion and singlet oxygen. J Clin Biochem Nutr. 2010;46(3):224–8. 10.3164/jcbn.09-84. 20490317 10.3164/jcbn.09-84 PMC2872227

[27] Allan LL. Physiological and pathological changes in skin from sunburn and suntan. JAMA. 1960;173(11):1227–31. 10.1001/jama.1960.73020290008011. 14418319 10.1001/jama.1960.73020290008011

[28] Law MH, Medland SE, Zhu G, et al. Genome-wide association shows that pigmentation genes play a role in skin aging. J Invest Dermatol. 2017;137(9):1887–94. 10.1016/j.jid.2017.04.026. 28502801 10.1016/j.jid.2017.04.026

[29] Chen Y, André M, Adhikari K, et al. A genome-wide association study identifies novel gene associations with facial skin wrinkling and mole count in Latin Americans. Br J Dermatol. 2021;185(5):988–98. 10.1111/bjd.20436. 33959940 10.1111/bjd.20436

[30] Buranasirin P, Pongpirul K, Meephansan J. Development of a global subjective skin aging assessment score from the perspective of dermatologists. BMC Res Notes. 2019;12(1):364. 10.1186/s13104-019-4404-z. 31253172 10.1186/s13104-019-4404-z PMC6599371

[31] Tschachler E, Morizot F. Ethnic differences in skin aging. In: Skin Aging. Springer-Verlag; 2006:23–31. 10.1007/3-540-32953-6_3.

[32] Vierkötter A, Hüls A, Yamamoto A, et al. Extrinsic skin ageing in German, Chinese and Japanese women manifests differently in all three groups depending on ethnic background, age and anatomical site. J Dermatol Sci. 2016;83:219–25. 10.1016/j.jdermsci.2016.05.011. 27289339 10.1016/j.jdermsci.2016.05.011

[33] Eun HC. Cutaneous photodamage in Asians. J Dermatol. 2001;28(11):614–6. 10.1111/j.1346-8138.2001.tb00045.x. 11770717 10.1111/j.1346-8138.2001.tb00045.x

[34] Halder RM. The role of retinoids in the management of cutaneous conditions in blacks. J Am Acad Dermatol. 1998;39(2):S98–103. 10.1016/s0190-9622(98)70455-4. 9703134 10.1016/s0190-9622(98)70455-4

[35] Halder RM, Grimes PE, McLaurin CI, Kress MA, Kenney JA. Incidence of common dermatoses in a predominantly black dermatologic practice. Cutis. 1983;32(4):388–390. https://pubmed.ncbi.nlm.nih.gov/6226496/. Accessed September 8, 2020. 6226496

[36] Hillebrand GG, Miyamoto K, Schnell B, Ichihashi M, Shinkura R, Akiba S. Quantitative evaluation of skin condition in an epidemiological survey of females living in northern versus southern Japan. J Dermatol Sci. 2001;27(SUPPL. 1):42–52. 10.1016/s0923-1811(01)00118-9. 10.1016/s0923-1811(01)00118-9 11514124

[37] Ng JY, Min X, Ng GY, Yi Q, Wong A, Chew FT. Dietary interventions in skin ageing: a systematic review and meta‑analysis. J Physiol Anthropol. 2025;44(26):1–25. 10.1186/s40101-025-00408-4. 41174715 10.1186/s40101-025-00408-4 PMC12577306

