# Supplementary figures and images for: A genome-wide association study identifies that SAMD5 interacts with regular Sun exposure to influence nasolabial folds development
Source: J Physiol Anthropol. 2026 Feb 28;45:10. doi: 10.1186/s40101-026-00423-z (PMC13059621; doi:10.1186/s40101-026-00423-z)

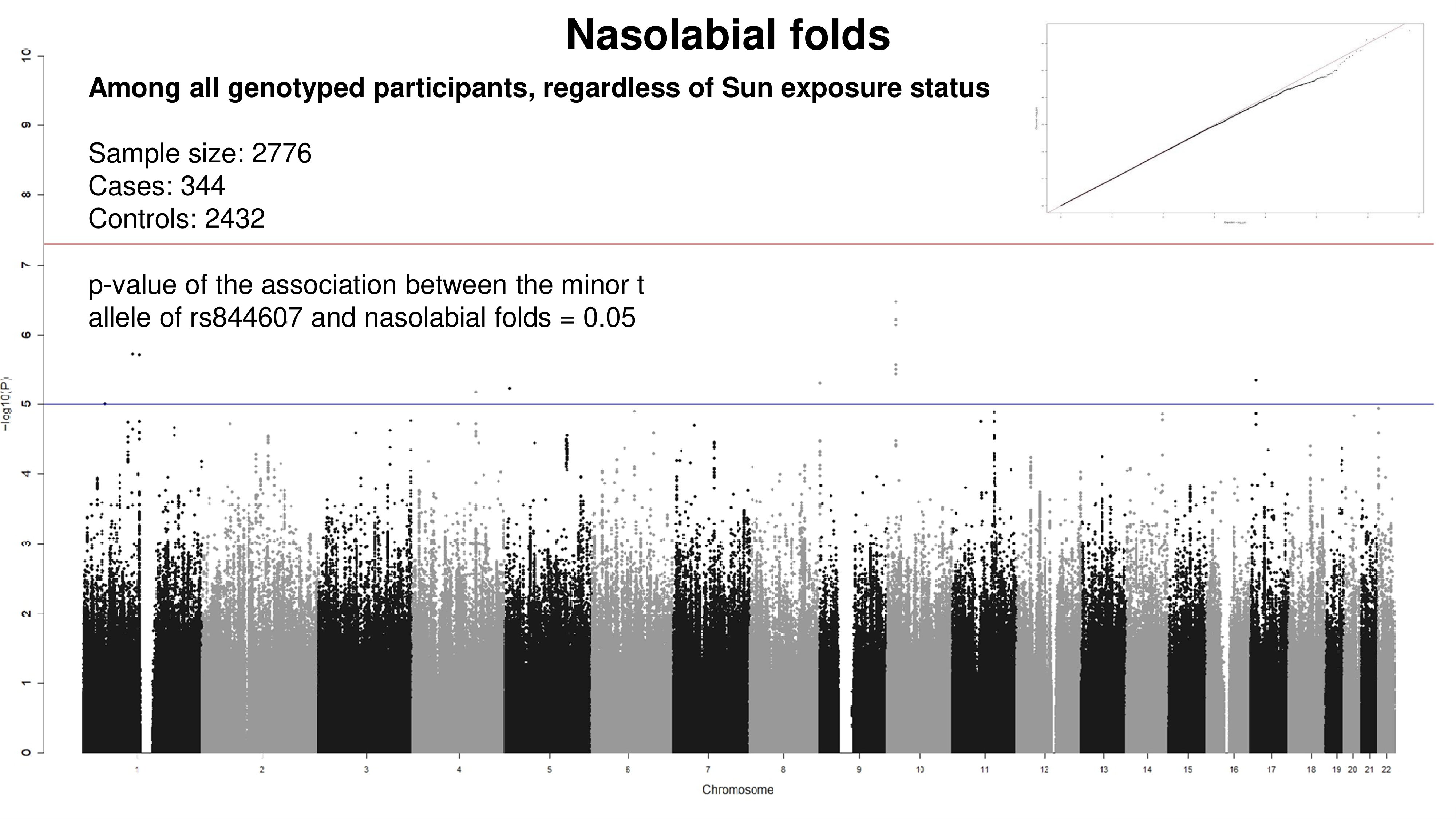

Supplement: Supplementary file 1 — Additional file 1: Genome-wide association study (GWAS) of nasolabial folds in participants with regular Sun exposure from the Singapore/Malaysia Cross-sectional Genetics Epidemiology Study (SMCGES). This Manhattan plot, generated using the qqman package on R with the RStudio interface in RStudio version 4.3.1, shows the genome-wide distribution of single nucleotide polymorphism (SNP) associations with nasolabial folds among all genotyped participants, regardless of Sun exposure status (n = 2776; cases = 344, controls = 2432). Each point represents an SNP, with chromosomal position on the x-axis and the corresponding –log10(p-value) on the y-axis. The blue horizontal line denotes the suggestive significance threshold (p = 1 × 10^–5^), while the red line marks the genome-wide significance threshold (p = 5 × 10^–8^). A clear drag of association signals is observed on chromosome 6 near the SAMD5 locus, with the lead SNP rs844607 exhibiting the strongest association; the minor t allele is associated with nasolabial folds, with an adjusted odds ratio (AOR) [95% confidence interval (CI)] = 1.21 [1.00–1.47], p = 0.05. The GWAS included 3,565,291 SNP variants genotyped from buccal cell DNA of 2,776 Chinese participants. The analysis was adjusted for age, sex, and the first three genetic principal components (PCs). In the top-right corner, the quantile–quantile (QQ) plot compares the observed –log10(p-values) (y-axis) to the expected –log10(p-values) under the null hypothesis (x-axis), with the red diagonal line representing the expected null distribution and black dots representing the observed data; the close correspondence between the two indicates that the test statistics follow the expected null distribution, with deviation at the upper tail consistent with true associations rather than systematic bias. [file 40101_2026_423_MOESM1_ESM.png]

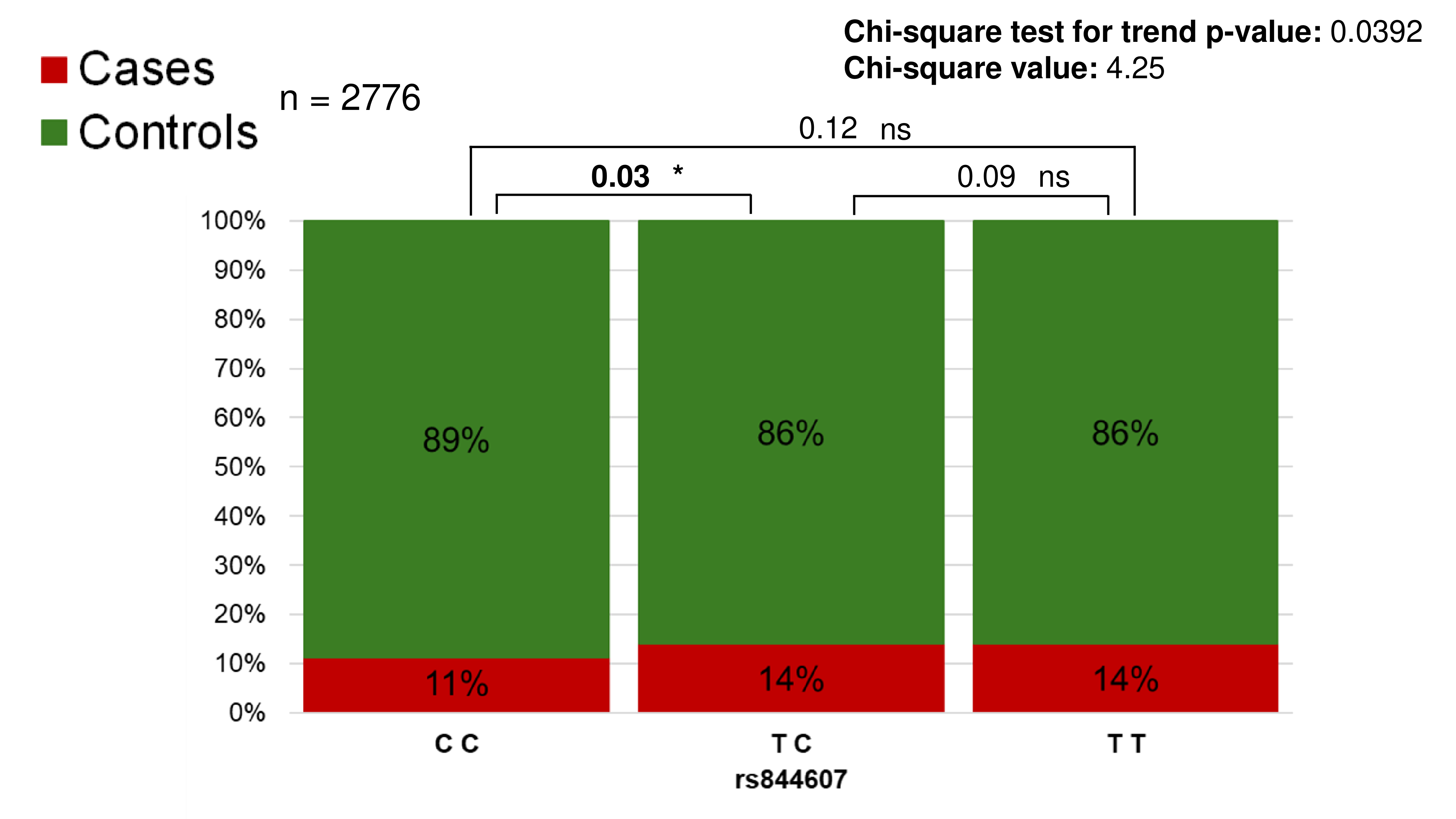

Supplement: Supplementary file 2 — Additional file 2: Association of rs844607 genotype with the prevalence of nasolabial folds among all genotyped participants from the Singapore/Malaysia Cross-sectional Genetics Epidemiology Study (SMCGES) regardless of Sun exposure status. This figure presents a 100% stacked bar chart illustrating the proportional distribution of nasolabial fold cases and controls across the three genotypes of rs844607 (CC, TC, TT). The x-axis represents the genotype categories, while the y-axis indicates the percentage of participants (0 to 100%) within each genotype group. The major allele is C and the minor allele is t. An additive trend is observed, with the proportion of nasolabial fold cases increasing stepwise from 11% in CC homozygotes, to 14% in TC heterozygotes, and to 14% in TT homozygotes. The χ2 test for trend (Extended Mantel–Haenszel Chi-square) demonstrates a significant dose-dependent relationship between the minor t allele and nasolabial folds (χ2 = 4.25, p = 0.0392). The χ2 test p-values for the case/control distributions in the three genotypes are 0.03 (CC vs TC), 0.09 (TC vs TT), and 0.12 (CC vs TT). The total sample size is n = 2776. Abbreviations: χ2 test, chi-square test; SMCGES, Singapore/Malaysia Cross-sectional Genetics Epidemiology Study. [file 40101_2026_423_MOESM2_ESM.png]

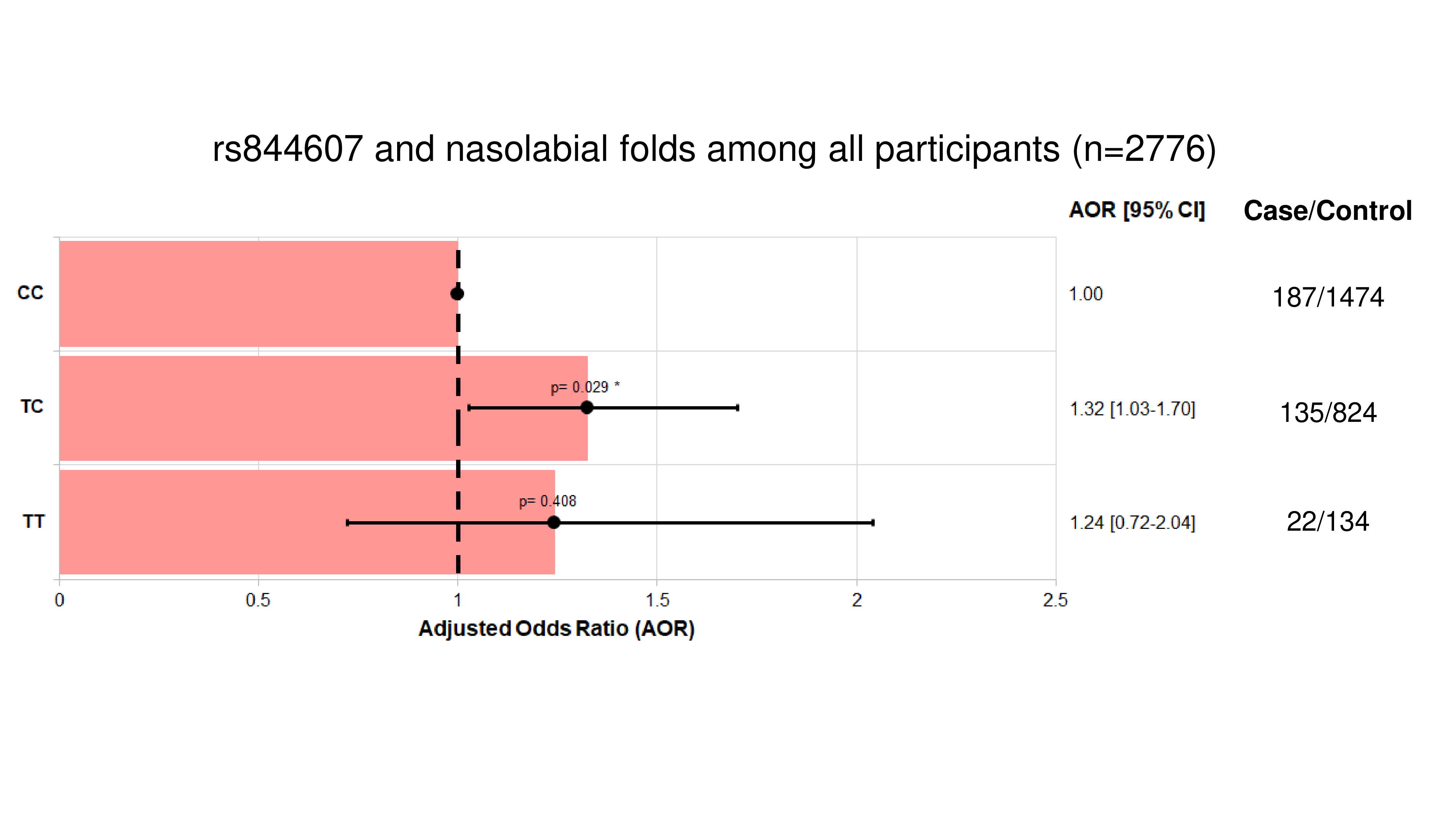

Supplement: Supplementary file 3 — Additional file 3: Adjusted odds ratios (AORs) for nasolabial folds according to rs844607 genotypes among all genotyped participants from the Singapore/Malaysia Cross-sectional Genetics Epidemiology Study (SMCGES) regardless of Sun exposure status. This bar chart displays the adjusted odds ratios for nasolabial folds across the three genotypes of rs844607 (CC, TC, TT) in all genotyped participants (n = 2776). The major allele is C and the minor allele is t. The genotype CC serves as the reference group (AOR = 1.00), indicated by a black dotted horizontal line at AOR = 1.00. Each bar represents the AOR with corresponding 95% confidence intervals (CIs) shown as solid black lines capped with vertical bars, and the point estimates are denoted by solid black circles. The adjusted p-value is displayed directly above each mean estimate. Compared with CC, individuals with TC exhibit a significantly higher odds of nasolabial folds with an AOR [95% CI] = 1.32 [1.03–1.70], p-value = 0.029. Compared with CC, individuals with TT exhibit a higher odds of nasolabial folds with an AOR [95% CI] = 1.24 [0.72–2.04], p-value = 0.408 after adjustment for age and sex. The case/control counts for each genotype are 187/1474 for CC, 135/824 for TC, and 22/134 for TT, indicating a stepwise increase in odds with each additional copy of the minor t allele. Abbreviations: AOR, adjusted odds ratio; CI, confidence interval; SMCGES, Singapore/Malaysia Cross-sectional Genetics Epidemiology Study. [file 40101_2026_423_MOESM3_ESM.png]

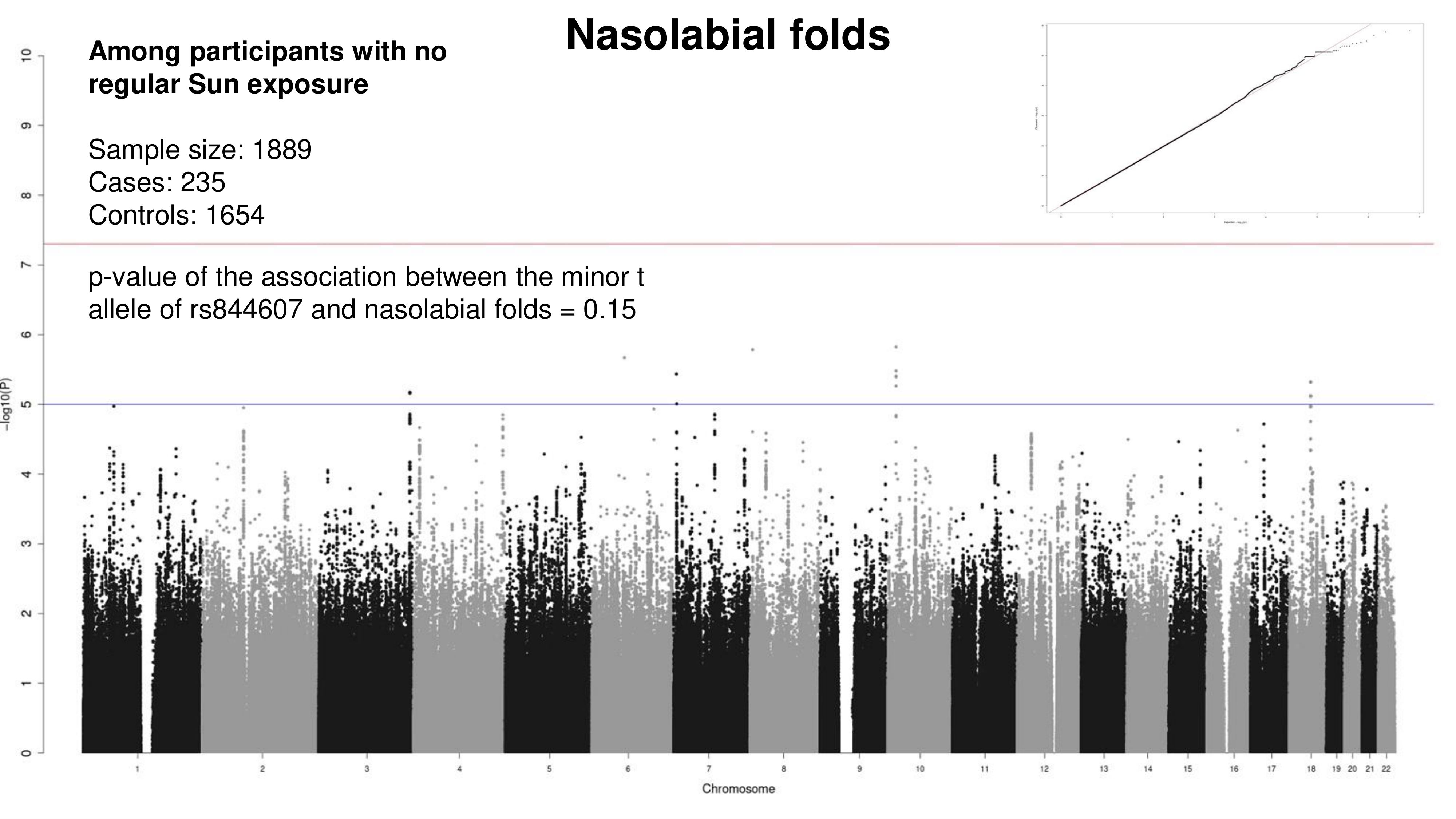

Supplement: Supplementary file 4 — Additional file 4: Genome-wide association study (GWAS) of nasolabial folds in participants with regular Sun exposure from the Singapore/Malaysia Cross-sectional Genetics Epidemiology Study (SMCGES). This Manhattan plot, generated using the qqman package on R with the RStudio interface in RStudio version 4.3.1, shows the genome-wide distribution of single nucleotide polymorphism (SNP) associations with nasolabial folds among participants reporting no regular Sun exposure (n = 1889; cases = 235, controls = 1654). Each point represents an SNP, with chromosomal position on the x-axis and the corresponding –log10(p-value) on the y-axis. The blue horizontal line denotes the suggestive significance threshold (p = 1 × 10^–5^), while the red line marks the genome-wide significance threshold (p = 5 × 10^–8^). A clear drag of association signals is observed on chromosome 6 near the SAMD5 locus, with the lead SNP rs844607 exhibiting the strongest association; the minor t allele is associated with nasolabial folds, with an adjusted odds ratio (AOR) [95% confidence interval (CI)] = 0.83 [0.65–1.07], p = 0.15. The GWAS included 3,565,291 SNP variants genotyped from buccal cell DNA of 1,889 Chinese participants. The analysis was adjusted for age, sex, and the first three genetic principal components (PCs). In the top-right corner, the quantile–quantile (QQ) plot compares the observed –log10(p-values) (y-axis) to the expected –log10(p-values) under the null hypothesis (x-axis), with the red diagonal line representing the expected null distribution and black dots representing the observed data; the close correspondence between the two indicates that the test statistics follow the expected null distribution, with deviation at the upper tail consistent with true associations rather than systematic bias. [file 40101_2026_423_MOESM4_ESM.png]

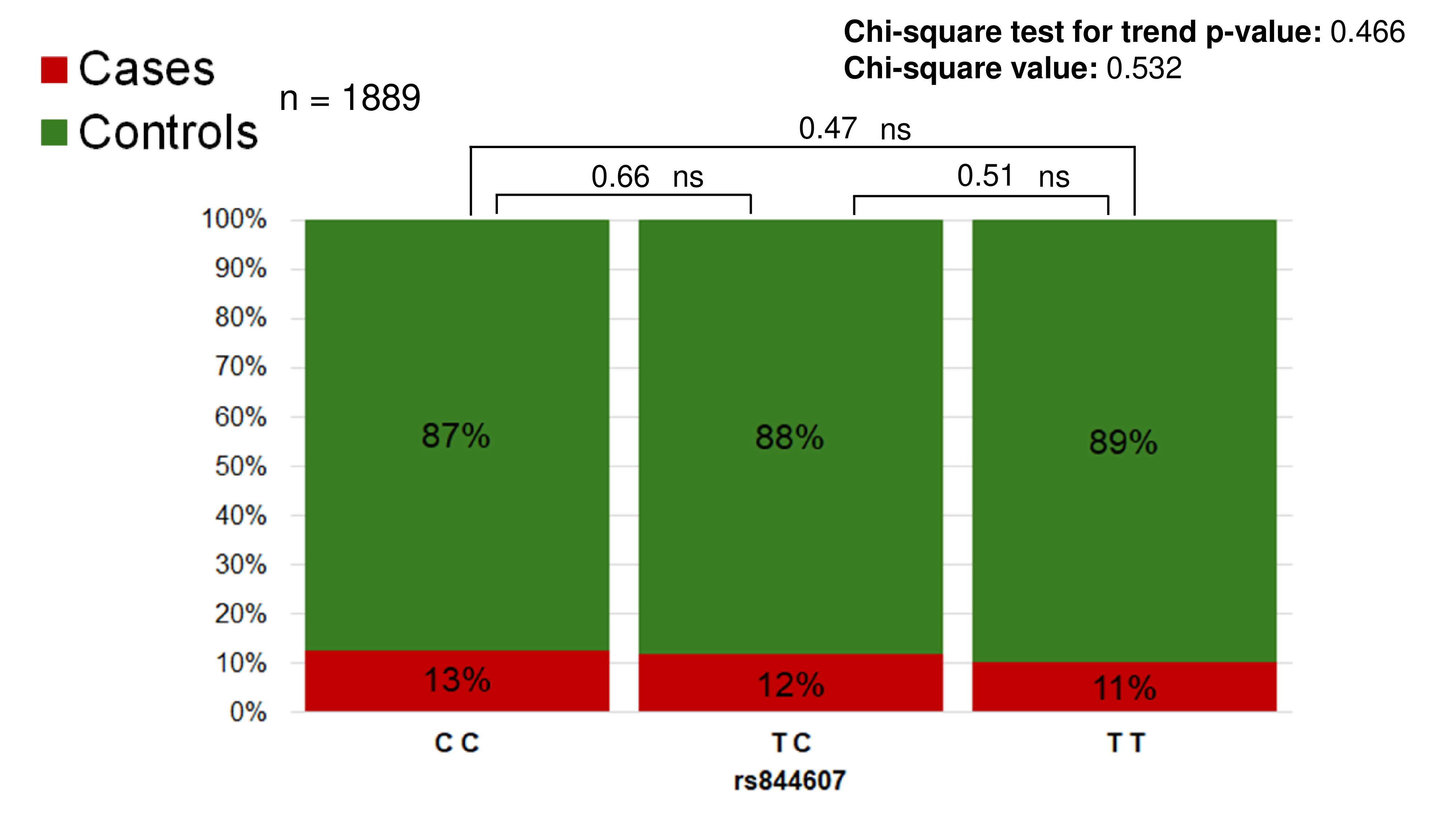

Supplement: Supplementary file 5 — Additional file 5: Association of rs844607 genotype with the prevalence of nasolabial folds among participants with no regular Sun exposure from the Singapore/Malaysia Cross-sectional Genetics Epidemiology Study (SMCGES). This figure presents a 100% stacked bar chart illustrating the proportional distribution of nasolabial fold cases and controls across the three genotypes of rs844607 (CC, TC, TT). The x-axis represents the genotype categories, while the y-axis indicates the percentage of participants (0 to 100%) within each genotype group. The major allele is C and the minor allele is t. No trend is observed. The proportion of nasolabial fold cases is 13% in CC homozygotes, 12% in TC heterozygotes, and to 11% in TT homozygotes. The χ2 test for trend (Extended Mantel–Haenszel Chi-square) demonstrates no significant dose-dependent relationship between the minor t allele and nasolabial folds (χ2 = 0.532, p = 0.466). The χ2 test p-values for the case/control distributions in the three genotypes are 0.66 (CC vs TC), 0.51 (TC vs TT), and 0.47 (CC vs TT). The total sample size is n = 1889. Abbreviations: χ2 test, chi-square test; SMCGES, Singapore/Malaysia Cross-sectional Genetics Epidemiology Study. [file 40101_2026_423_MOESM5_ESM.png]

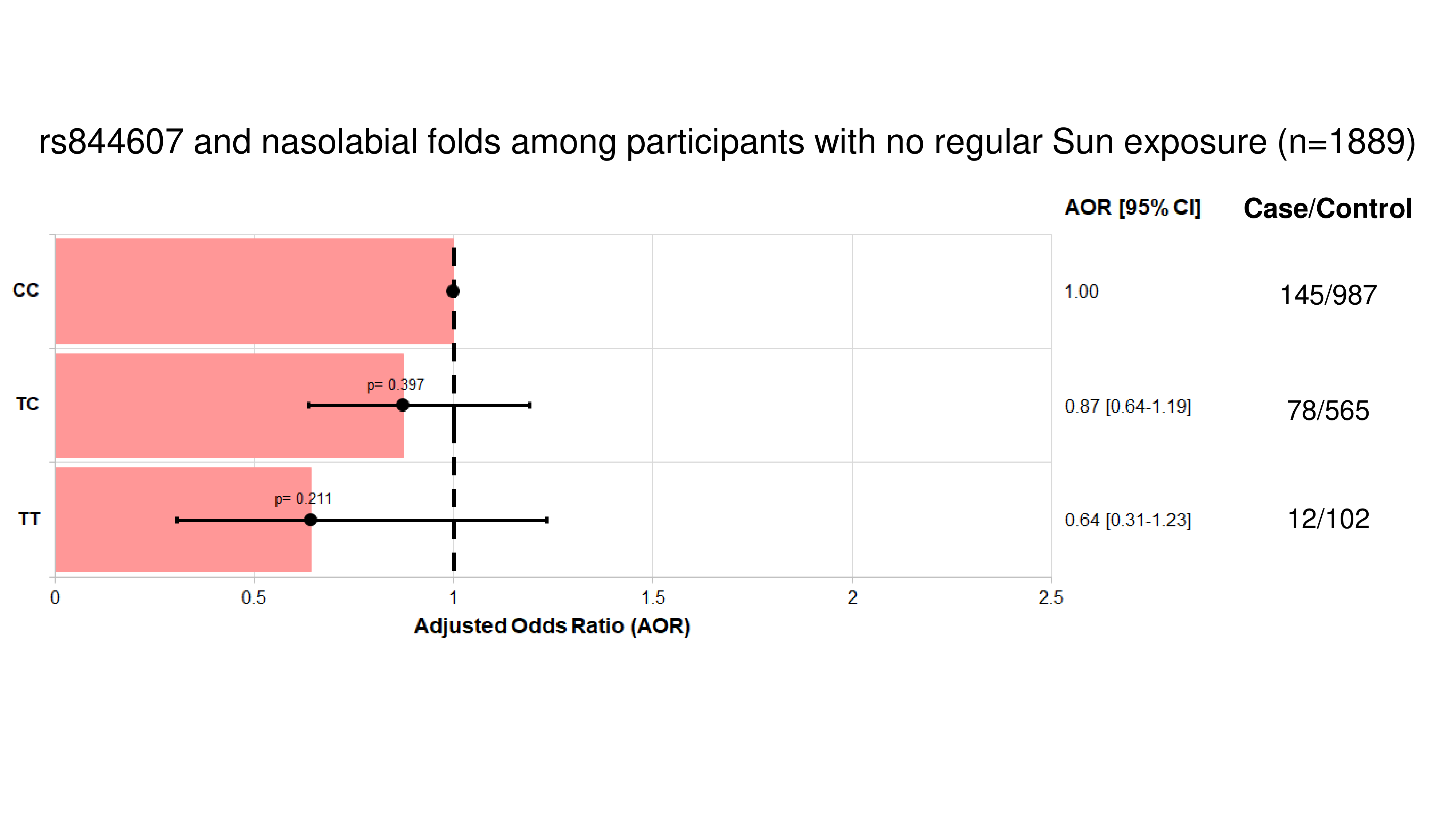

Supplement: Supplementary file 6 — Additional file 6: Adjusted odds ratios (AORs) for nasolabial folds according to rs844607 genotypes among participants with no regular Sun exposure from the Singapore/Malaysia Cross-sectional Genetics Epidemiology Study (SMCGES). This bar chart displays the adjusted odds ratios for nasolabial folds across the three genotypes of rs844607 (CC, TC, TT) in all genotyped participants (n = 1889). The major allele is C and the minor allele is t. The genotype CC serves as the reference group (AOR = 1.00), indicated by a black dotted horizontal line at AOR = 1.00. Each bar represents the AOR with corresponding 95% confidence intervals (CIs) shown as solid black lines capped with vertical bars, and the point estimates are denoted by solid black circles. The adjusted p-value is displayed directly above each mean estimate. Compared with CC, individuals with TC exhibit a lower odds of nasolabial folds with an AOR [95% CI] = 0.87 [0.64–1.19], p-value = 0.397. Compared with CC, individuals with TT exhibit a lower odds of nasolabial folds with an AOR [95% CI] = 0.64 [0.31–1.23], p-value = 0.211 after adjustment for age and sex. The case/control counts for each genotype are 145/987 for CC, 78/565 for TC, and 12/102 for TT, indicating a stepwise increase in odds with each additional copy of the minor t allele. Abbreviations: AOR, adjusted odds ratio; CI, confidence interval; SMCGES, Singapore/Malaysia Cross-sectional Genetics Epidemiology Study. [file 40101_2026_423_MOESM6_ESM.png]
